# Alzheimer's Disease Blood Biomarkers Associated With Neuroinflammation as Therapeutic Targets for Early Personalized Intervention

**DOI:** 10.3389/fdgth.2022.875895

**Published:** 2022-07-11

**Authors:** Sher Li Oh, Meikun Zhou, Eunice W. M. Chin, Gautami Amarnath, Chee Hoe Cheah, Kok Pin Ng, Nagaendran Kandiah, Eyleen L. K. Goh, Keng-Hwee Chiam

**Affiliations:** ^1^Lee Kong Chian School of Medicine, Nanyang Technological University, Singapore, Singapore; ^2^IGP-Neuroscience, Interdisciplinary Graduate Programme, Nanyang Technological University, Singapore, Singapore; ^3^Bioinformatics Institute, A^*^STAR, Singapore, Singapore; ^4^Department of Neurology, National Neuroscience Institute, Singapore, Singapore; ^5^Duke-NUS Medical School, Singapore, Singapore

**Keywords:** Alzheimer's Disease, mild cognitive impairment, neurodegeneration, biomarkers, machine learning, gene expression

## Abstract

The definitive diagnosis of Alzheimer's Disease (AD) without the need for neuropathological confirmation remains a challenge in AD research today, despite efforts to uncover the molecular and biological underpinnings of the disease process. Furthermore, the potential for therapeutic intervention is limited upon the onset of symptoms, providing motivation for studying and treating the AD precursor mild cognitive impairment (MCI), the prodromal stage of AD instead. Applying machine learning classification to transcriptomic data of MCI, AD, and cognitively normal (CN) control patients, we identified differentially expressed genes that serve as biomarkers for the characterization and classification of subjects into MCI or AD groups. Predictive models employing these biomarker genes exhibited good classification performances for CN, MCI, and AD, significantly above random chance. The PI3K-Akt, IL-17, JAK-STAT, TNF, and Ras signaling pathways were also enriched in these biomarker genes, indicating their diagnostic potential and pathophysiological roles in MCI and AD. These findings could aid in the recognition of MCI and AD risk in clinical settings, allow for the tracking of disease progression over time in individuals as part of a therapeutic approach, and provide possible personalized drug targets for early intervention of MCI and AD.

## Introduction

Alzheimer's Disease (AD) is one of the most widely studied neurodegenerative disorders, and is associated with widespread brain atrophy and cognitive decline. It is clinically characterized by memory deficits, and patients develop progressive neuropsychiatric symptoms such as apathy, delusions, and agitation (1). The World Health Organization (WHO) estimates that AD is the most common form of dementia and that it stands to be the seventh leading cause of death in elderly worldwide. The neuropathology of AD is widely associated with the accumulation of amyloid-beta (Aβ) plaques and neurofibrillary tangles in the brain, and the definitive diagnosis of AD is only possible by post-mortem microscopic examination of brain tissues (2). While neuropathologic changes are well correlated with cognitive decline in AD (3), it remains unfeasible to examine brain tissue for AD diagnosis in clinical settings.

Diagnostic approaches for AD historically involve the interpretation of neuroimaging data, neuropsychological tests and laboratory tests (4). Although much progress has been made in the diagnosis of AD, the methodology of diagnosis relies heavily on clinicians' interpretations of laboratory results and neuropsychological tests for detecting cognitive deficits (5), with cerebrospinal fluid biomarkers such as phosphorylated tau only being recently included as considerations for clinical diagnosis (6). However, the reliability of AD diagnosis remains variable due to several confounders such as human interpretation, age and education. For instance, the Mini-Mental State Exam (MMSE) is widely used by clinicians for the screening of dementia by administering a 30-points questionnaire to evaluate a subject's orientation, recall, attention, language, and comprehension abilities. While the MMSE is a quick way of assessing subjects' cognitive decline, its use in monitoring the progression of AD is limited due to its low sensitivity to intermediate conditions such as mild cognitive impairment (MCI) (7). The complexity due to the different variants of AD also requires clinicians to evaluate a subject's condition by relying on a combination of other non-quantitative factors for diagnosis, which can include, but are not limited to, the medical history of patients, neuroimaging, and interviewing patients' kin (8). This complex combination of diagnostic factors often leads to variability in diagnosis by different clinicians, therefore a diagnostic approach that is based on quantitative measurements of the biological process of AD will be useful to provide more timely and accurate AD screening.

Recent advancements in genome sequencing technology have contributed to improved accessibility of large transcriptomic datasets, leading to greater opportunities for identifying biomarkers associated with complex and rare diseases (9). The quantification of biomarkers from gene expression profiles may be a possible diagnostic approach for AD that reduces reliance on clinicians' experience thereby reducing human error and eliminating subjectivity in diagnosis. Gene expression profiles from several studies have uncovered valuable patterns in AD patients, namely the presence of Aβ and hyperphosphorylated tau in the brain (10). Unfortunately, such approaches still face the limitation of using brain tissue from biopsies, which carries the risk of complications (11), and may not be translatable to all clinical settings. Alternatively, blood gene expression profiles may prove useful in AD screening with significant reduction of risks and greater tissue accessibility. Two large-scale blood gene expression datasets were conducted in recent years that aim to detect biomarkers for early diagnosis of AD: the Alzheimer's Disease Neuroimaging Initiative [ADNI; (12)] and AddNeuroMed (13, 14), which include subjects from North America and Europe respectively Both studies comprise similar protocols and data modalities, namely clinical and cognitive tests, blood transcriptomics, and neuroimaging, although the ADNI includes additional data from positron emission tomography imaging and from genetic studies on cerebrospinal fluid biomarkers of AD (15).

In this study, we identified gene expression profiles associated with inflammation, vascular dementia, MCI, AD, stroke and other cerebrovascular diseases in a Singaporean MCI and AD cohort. The focus on a largely inflammatory set of genes stems from earlier work suggesting that neuroinflammation in AD is closely linked to neurodegeneration, the severity of which can be observed as alterations in white matter hyperintensity through neuroimaging. We therefore aim to identify potential biomarkers that could predict neuroinflammatory changes before severe and irreversible neurodegeneration in AD, or even in MCI, so as to provide early intervention. Our findings were then validated using blood gene expression data from ADNI.

However, blood gene expression studies are limited by two main drawbacks. Firstly, the gene expression profiles are usually difficult to interpret due to the data being highly variable (16). Secondly, the large proportion of genes to a small proportion of subjects creates a statistical hurdle in the search for potential biomarkers. Technological advancements in recent years have provided various supervised and unsupervised models for the applications in features extraction of large expression datasets (17). Supervised machine learning models, such as Random Forests (RF), are particularly useful in such applications due to their flexibility in both classification and regression studies. RF also has the advantage of tackling the non-linear nature of gene expression profiles (18). In this study, we employed a multi-stage machine learning pipeline for the exploration of selecting differentially expressed genes (DEGs) in AD and MCI subjects. The analysis pipeline was built upon Boruta, a statistically driven machine learning method, which differentiated the important genes from noise after rigorous iterations of RF models (19). We showed that machine learning techniques may prove useful in discovering potential biomarkers for AD and MCI detection in the large data pool of blood gene expressions.

## Materials and Methods

**Figure 1:**
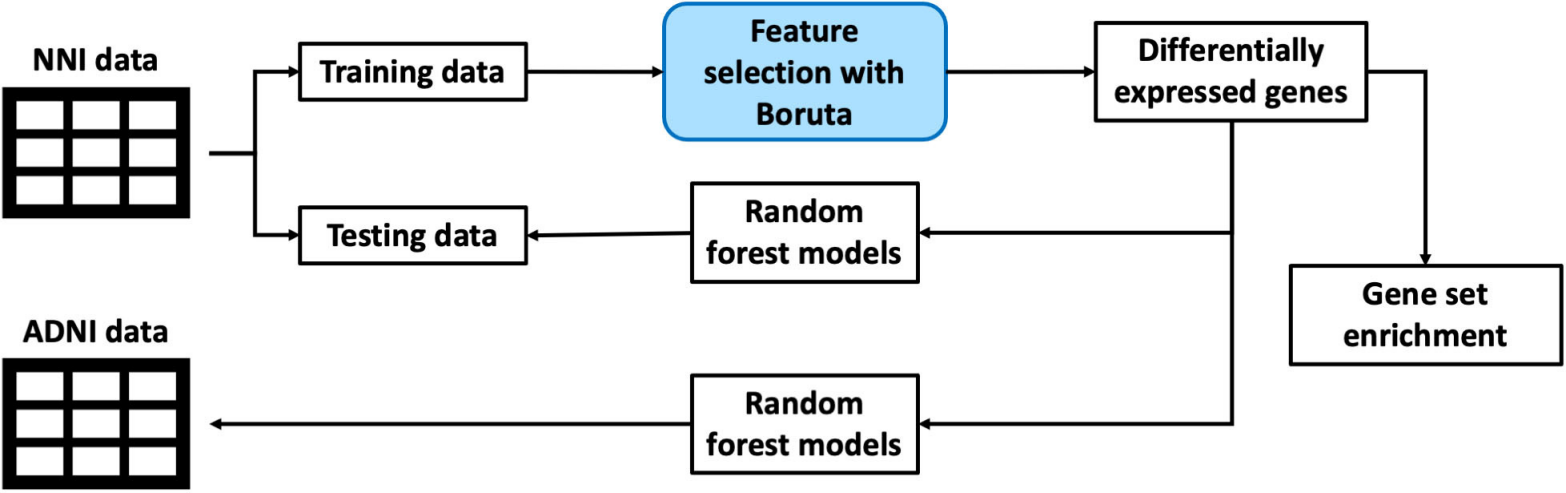
An illustration of the overall framework for studying biomarkers from blood gene expression.

This section discusses the methodology used for processing of datasets as well as machine learning pipeline used to extract out the genes of interest and building a classification model. The overall framework is illustrated in a simplified diagram as shown in Figure 1.

### Study Subjects and Datasets

This study was conducted on blood gene expression datasets consisting of one set of clinical samples recruited from the Memory clinic of the National Neuroscience Institute in Singapore (NNI), and the publicly available ADNI dataset (12). The distribution of subjects from each dataset is shown in Table 1.

**Table 1:** Demographics and Mini-Mental State Examination (MMSE) scores of cognitively normal (CN) controls, mild cognitive impairment (MCI), and Alzheimer's Disease (AD) subjects in the Alzheimer's Disease Neuroimaging Initiative (ADNI) and National Neuroscience Institute (NNI) datasets.

	**CN**	**MCI**	**AD**	**Total**
**NNI**				
Number of subjects (%)	99 (39.0%)	61 (24.0%)	94 (37.0%)	254 (100%)
Age (mean ± s.e.)	63.35 ± 0.69	65.03 ± 0.86	71.18 ± 0.84	66.65 ± 0.51
Gender	42 male, 57 female	24 male, 37 female	48 male, 46 female	114 male, 140 female
MMSE (mean ± s.e.)	28.62 ± 0.15	27.49 ± 0.20	21.91 + 0.48	25.87 ± 0.27
**ADNI**				
Number of subjects (%)	261 (35.1%)	439 (59.0%)	44 (5.9%)	744 (100%)
Age (mean ± s.e.)	75.56 ± 0.39	72.53 ± 0.38	75.20 ± 1.43	73.75 ± 0.28
Gender	125 male, 136 female	256 male, 183 female	27 male, 17 female	408 male, 336 female
MMSE (mean ± s.e.)	27.81 ± 0.21	24.51 ± 0.30	18.95 ± 0.82	25.34 ± 0.21

*CN, cognitively normal; MCI, mild cognitive impairment; AD, Alzheimer's Disease*.

In this investigation, the NNI dataset was used as the discovery cohort for the identification of differentially expressed genes as potential biomarkers for MCI and AD, and their respective biological pathways between CN, MCI, and AD subjects. The applicability of these genes to the identification of MCI and AD subjects was then evaluated on the ADNI dataset.

Clinical samples from NNI were collected over a period of 3 years, from 2013 to 2016. Informed consent was obtained for all subjects. Participants underwent clinical evaluation of psychological and cognitive performance, using the MMSE, the Montreal Cognitive Assessment (MoCA), and magnetic resonance imaging (MRI). Healthy control (CN) subjects were further required to have no cognitive complaints, no significant cognitive defects, and a clinical dementia rating (CDR) of 0. Diagnosis of MCI was based on the criteria of the National Institute on Aging–Alzheimer's Association (NIA-AA) Research Framework (20), the clinical presentation of cognitive symptoms and neuropsychological deficits without significant functional impairment, and a CDR score of 0.5. Subjects with AD were diagnosed using criteria from the National Institute of Neurological and Communicative Disorders and Stroke (NINCDS) and the Alzheimer's Disease and Related Disorders Association (ADRDA). According to the NINCDS-ADRDA criteria, patients with mild AD display cognitive symptoms and deficits according to neuropsychological evaluation, with significant functional impairment (4). Patients with mild AD were also defined as having a CDR score of 1. The NNI data therefore constitute 254 subjects (99 CN, 61 MCI, and 94 AD, listed in Table 1).

### RNA Isolation and Quantification for NNI Data

Non-fasting venous blood was drawn via antecubital venipuncture. RNA was isolated from 1 ml of whole blood using the QIAamp RNA Blood Mini Kit (QIAGEN no. 52304). Total RNA from subjects was extracted from whole blood on the same day as blood collection. Isolated RNA was stored at −80°C. Complementary DNA (cDNA) was obtained by reverse transcription of 10 ng of total RNA (Fluidigm no. 100-6298). One hundred eighty two target genes were shortlisted from a literature search of genes associated with inflammatory response, vascular dementia, MCI, AD, stroke, and other cerebrovascular diseases (Supplementary Table 1). Primers for the gene targets were designed using Primer3 (21) or with reference to the OriGene database, and validated by real time quantitative polymerase chain reaction (qPCR) using RNA isolated from lipopolysaccharide-induced human lymphoblastoid cells. The 96.96 Dynamic Array Integrated Fluidic Circuits (IFC) for Gene Expression was used for quantifying RNA for each gene. The data was collected using the BioMark HD Image Capture System for further analysis.

### Data Preparation and Pre-processing

Blood gene expression data from NNI were normalized against the housekeeping gene *RP2* to obtain relative expression values of each gene. Replicates of samples were averaged to obtain a single gene expression value per gene. Genes with more than 25% missing values were removed from the analysis. For the remaining genes, missing value imputation was carried out by the adaptive LSimpute method using the missMethods package (v.0.4.0) in R (v.4.1.1), based on the least squares principle and correlations between genes and between arrays (22–24). This resulted in expression data from 176 genes for analysis in the NNI dataset (Supplementary Table 1).

Likewise, relative gene expression values for the ADNI dataset were obtained by normalizing all gene expression values, which had been obtained by microarray, by that of *RP2* for each respective subject, and relative expression values for replicate samples were averaged to obtain a final relative gene expression value for each subject. A subset of the ADNI dataset containing 151 genes in common with NNI was used for analysis. For both datasets, uniform manifold approximation and projection (UMAP) plots were constructed and visualized using the umap package (v.0.2.7.0) and plotly graphing library in R respectively (25, 26).

To verify that the NNI data were not strongly influenced by age and gender of the subjects, preliminary random forest regression models were constructed for regression of age, and for classification of male vs. female, and for CN vs. MCI, CN vs. AD, and MCI vs. AD, using the randomForest package (v.4.7.1) in R (27). The top 30 genes with greatest variable importance, quantified by their influence on the prediction error for each model, were denoted as being either age-dependent, gender-dependent, or diagnosis-dependent variables. The age-dependent and gender-dependent variables were compared against diagnosis-dependent variables to determine the proportion of genes whose expression values are potentially confounded by age and gender.

In preparation for feature selection, the NNI data were split into training and testing datasets. Data splits were conducted using the caTools package in R (v.1.17), such that each split preserves the original relative distributions of CN, MCI, and AD subjects in the training and testing data. For the identification of differentially expressed genes, 80% of the NNI data were used for training, while the remaining 20% were reserved as testing data for internal validation. A 80/20 training/testing split was also conducted on the ADNI data in preparation for external validation.

The unequal distribution of CN, MCI, and AD subjects in each dataset presented potential risks of increased bias during feature selection and classification. This was particularly pertinent for the ADNI data used in external validation, where the class imbalance caused by the higher proportion of MCI patients and relatively lower number of AD patients could have led to deceptively high accuracy metrics if the data were employed directly. To overcome this, the Synthetic Minority Oversampling Technique (SMOTE) was applied on the training and testing data from both NNI and ADNI (28), employing functions from the smotefamily package in R (v.1.3.1). SMOTE oversampled underrepresented groups by repeatedly generating a new data point between a randomly selected real data point, and a randomly selected point among its *k* nearest neighbors. In order to balance our training and testing data, for each underrepresented group, new data points were generated by SMOTE to equal the number of data points of the majority group, such that each class contained the same number of data points at each feature selection or classification step.

### Feature Selection for Differentially Expressed Genes

Differentially expressed genes (DEGs) were identified for each pairwise comparison, specifically CN vs. MCI, CN vs. AD, and MCI vs. AD. Boruta was employed to extract DEGs from the balanced training NNI data based on multiple iterations of the RF classifier (19). The RF classifier was selected for its quick performance and unbiasedness in classification through majority voting. The Boruta method selects for DEGs through a statistically rigorous approach by comparing the input features against shadow variables, derived from randomization of input variable values, and collating the variables that have greater feature importance than the best-performing shadow feature for subject classification at every run, denoted as a “hit” for that variable for that run. Over multiple runs, the number of hits for each variable makes up a binomial distribution, from which the list of important genes can be determined. To further eliminate variability, for each pairwise comparison, up to 1,000 runs were performed within each round of Boruta, and Boruta was itself conducted 10 times. Genes that were deemed important more than 50% of the time were defined as being differentially expressed between the two conditions.

### Validation and Evaluation of Biomarkers

Validation was conducted by constructing RF classifier models using the DEGs from Boruta for each pairwise comparison, which were trained on SMOTE-balanced data derived from the 80% training data of each dataset, and tested on the reserved 20% testing data, which was also balanced by SMOTE. Apart from internal validation on NNI data, to further validate that the DEGs identified from the NNI data were generalisable as biomarkers for MCI and AD, external validation was carried out by constructing RF classifier models from the NNI DEGs that were also present in ADNI data, and evaluating the performance of these models on ADNI data.

In both internal and external validation, the highest accuracies from 10 iterations of evaluation consisting of 30 RF classifier models each, were recorded and compared against 50% accuracy for each pairwise comparison using one-tailed Student's *t*-tests.

Protein-protein association networks were constructed based on DEGs from Boruta using the Search Tool for the Retrieval of Interacting Genes/Proteins (STRING) database [v.11.5; (29)].

### Gene Set Enrichment Analysis

The roles of the DEGs in a wider biological landscape were explored by annotating these genes with their Kyoto Encyclopedia of Genes and Genomes (KEGG) pathways (30), using the enrichR package (v.3.0) in R (31, 32). The KEGG pathways that overlap between the three pairwise comparisons were of particular interest as they could represent biological processes implicated during transitions from CN to MCI to AD.

## Results

### Heterogeneity in Alzheimer's Disease

**Figure 2:**
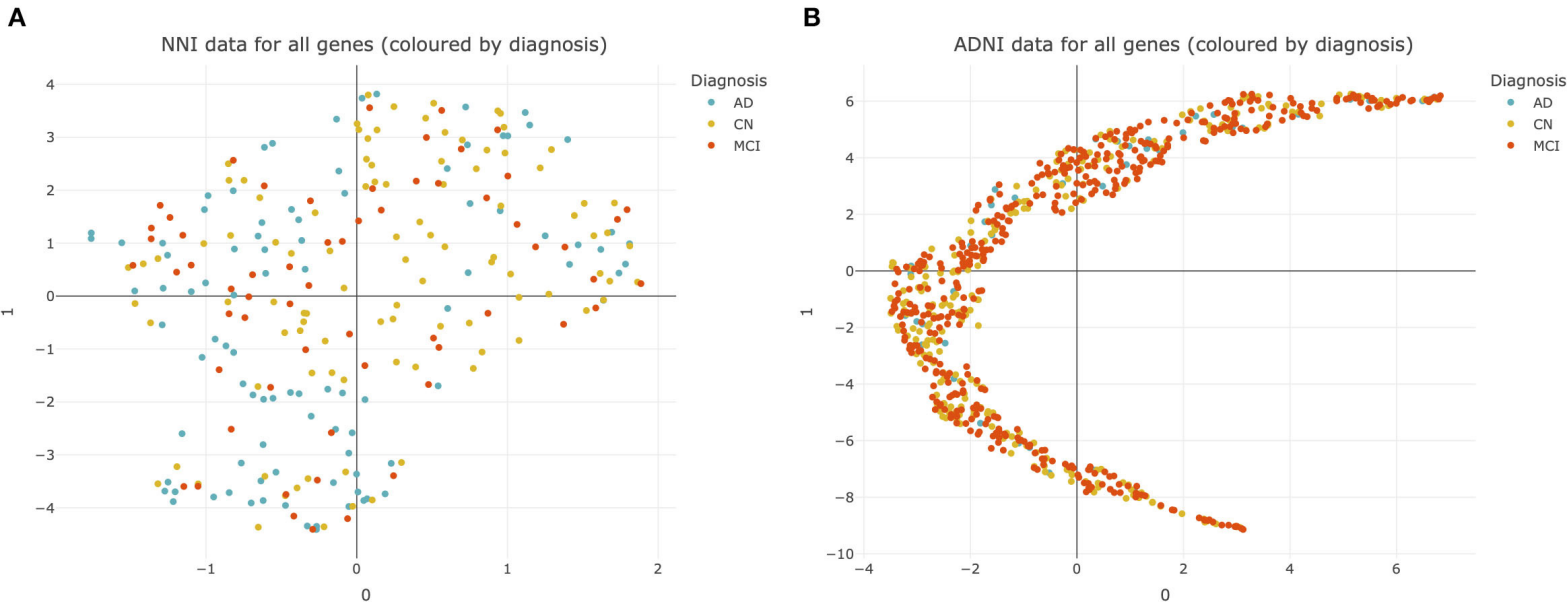
Uniform manifold approximation and projection (UMAP) plots of datasets used for analysis. **(A)** National Neuroscience Institute (NNI) data colored by diagnosis. Each point represents data from 176 genes from a single subject. **(B)** Alzheimer's Disease Neuroimaging Initiative (ADNI) data colored by diagnosis. Each point represents data from 20092 genes from a single subject. CN, cognitively normal; MCI, mild cognitive impairment; AD, Alzheimer's Disease.

Heterogeneity in AD presents difficulties for diagnosis and developing drug treatments (33). This heterogeneity was observed even at the transcriptomic level, as illustrated by UMAP plots of the NNI and ADNI datasets (Figure 2).

There was no clear separation between CN, MCI, and AD subjects within both the NNI and ADNI datasets (Figures 2A,B). This suggests that there is no clear cluster of genes that characterize MCI and AD to differentiate them from the CN subjects. This pattern was retained even after the NNI and ADNI datasets were split into training/testing sets and adjusted for class imbalance using SMOTE, demonstrating that the lack of clear clusters is not an effect of class imbalance and differences between each group of subjects cannot be easily resolved (Supplementary Figure 1).

The lack of clear defining delineations between groups of subjects further complicates diagnosis for these conditions, and demonstrates how there is no “one size fits all” approach for AD treatment, emphasizing the need for personalized treatments based on one's unique disease profile. Therefore, we employed the Boruta algorithm to identify differentially expressed genes and potential biomarkers within this complex dataset.

### Differentially Expressed Genes Identified as Potential Biomarkers

According to random forest regression models for age-dependent variables and classification models for gender and pairwise diagnosis conditions, more than 82% of diagnosis-dependent variables were not age-dependent, and 80% of diagnosis-dependent variables were not gender-dependent, suggesting that most differentially expressed genes across diagnosis in this study reflect changes across disease rather than age and gender differences between subjects.

From feature selection on the NNI training data balanced by SMOTE, 17 DEGs were identified between CN and MCI subjects, 10 genes identified between CN and AD subjects, and 16 genes identified between MCI and AD subjects, from a total of 176 genes in the initial dataset (Table 2).

**Table 2:** Differentially expressed genes from pairwise comparisons using National Neuroscience Institute (NNI) data.

**Comparison**	**Differentially expressed genes**
CN vs. MCI (17 genes)	ABCA7, CA4, CCL3, CD31, CSF1, F5, FGF2, TNNT2, IKBKG, IL17A, ITGB3, KITLG, LPA, NOS2, OSM, SF3B1, TBP
CN vs. AD (10 genes)	CBL, CCL18, CCL27, DNMT3A, FGF1, IL23, IL4R, NFKB1, THPO, TNFB
MCI vs. AD (16 genes)	CA4, CCL3, CCL4, CCL5, CCL7, CRP, CSF1, EDN1, F5, IL13, IL4R, IL6, IL7, NOS2, NOTCH3, OCLN

*This table shows the list of differentially expressed genes for each pairwise comparison. Boruta demonstrates that 17 genes are important for distinguishing between cognitively normal (CN) control vs. mild cognitive impairment (MCI) subjects, 10 genes are important for CN vs. Alzheimer's Disease (AD), and 16 genes are important for MCI vs. AD, respectively*.

These potential biomarker genes were used to construct predictive RF models for classifying the remaining 20% data from the NNI dataset balanced by SMOTE, as well as subjects from ADNI. Using the list of DEGs as predictive features to classify subjects of the ADNI dataset, the mean highest accuracies obtained were 59.55, 55.96, and 56.65% for the pairwise comparisons CN vs. MCI, CN vs. AD, and MCI vs. AD, respectively (Table 3). The classification accuracies using the list of DEGs as biomarkers were significantly higher than random classification of 50% for all three pairwise comparisons. Similarly, RF models constructed using the list of DEGs evaluated on NNI data classified between subjects from each pairwise comparison, i.e., CN vs. MCI, CN vs. AD, and MCI vs. AD, with max accuracies significantly higher than random classification (Supplementary Table 2).

**Table 3:** Highest prediction accuracy for Alzheimer's Disease Neuroimaging Initiative (ADNI) data using differentially expressed genes from National Neuroscience Institute (NNI) data as predictive features.

**Comparison**	**Highest prediction accuracy (mean % ±s.e.)**	***p*** **-value**
CN vs. MCI	59.55 ± 0.24	8.78e-12 ^***^
CN vs. AD	55.96 ± 0.13	2.46e-12 ^***^
MCI vs. AD	56.65 ± 0.09	2.79e-14 ^***^

*This table shows the mean and standard error (s.e.) of the highest prediction percent accuracy when random forest models constructed using differentially expressed genes shown in Table 2 are used to classify subjects between pairwise comparisons of cognitively normal (CN) control vs. mild cognitive impairment (MCI), CN vs. Alzheimer's Disease (AD), and MCI vs. AD, are used to classify subjects from ADNI*.

*** *indicates p < 0.001 according to a one-tailed t-test with the alternative hypothesis that the mean highest prediction percent accuracy is >50%*.

Several previously published studies aiming to identify blood biomarkers for AD focus on distinguishing between healthy and disease states, and demonstrate variations in DEGs between studies depending on the analysis methods employed (34, 35). In contrast, in addition to identifying DEGs associated only with AD, this investigation aims to identify genes that are differentially expressed in MCI, compared to CN and AD, specifically in the context of inflammatory genes. Nevertheless, we evaluated the performance of DEGs from Li et al. (34, 35) in pairwise classifications of CN vs. MCI, CN vs. AD, and MCI vs. AD, compared with the three sets of DEGs from this study. The DEGs identified in this study performed similarly to those from previous studies in classifying between CN and AD subjects (Supplementary Table 3). Our DEGs identified performed better in the classification of CN vs. MCI and MCI vs. AD subjects. Overall, pairwise classification using DEGs identified in this study is likely more representative of differential expression across CN, MCI, and AD, compared to genes in past studies that focus on AD classification.

**Figure 3:**
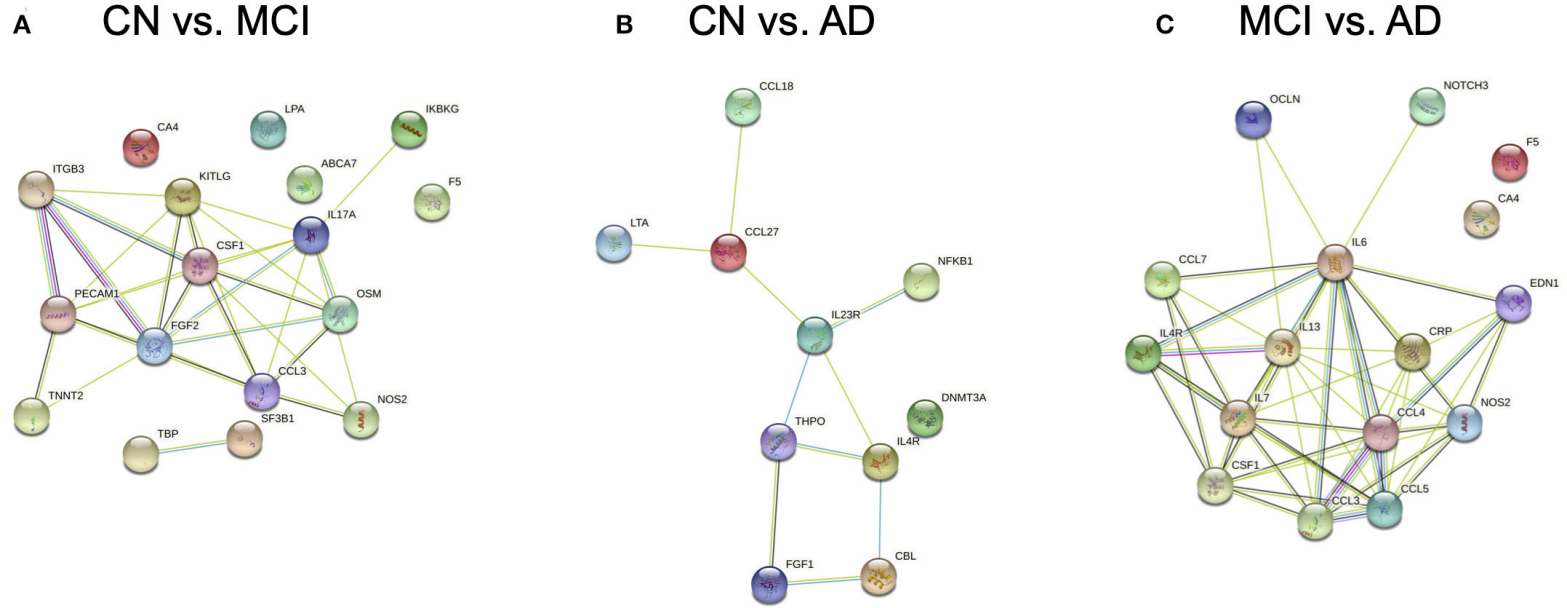
Protein-protein association networks constructed using the Search Tool for the Retrieval of Interacting Genes/Proteins (STRING) database. Proteins displayed are expressed from genes identified by Boruta from pairwise comparisons between **(A)** cognitively normal (CN) control and mild cognitive impairment (MCI), **(B)** CN and Alzheimer's Disease (AD), and **(C)** MCI and AD subjects.

Protein-protein association networks obtained by STRING demonstrated that most of the DEGs across the three pairwise comparisons have been shown or hypothesized to share protein functions in past studies, particularly for genes from CN vs. AD and MCI vs. AD (Figure 3). This indicates that DEGs identified by Boruta are functionally related, thus supporting the possibility that the biological regulation of protein networks involving these genes is affected during MCI and AD development.

### Differentially Expressed Pathways in MCI and AD

**Figure 4:**
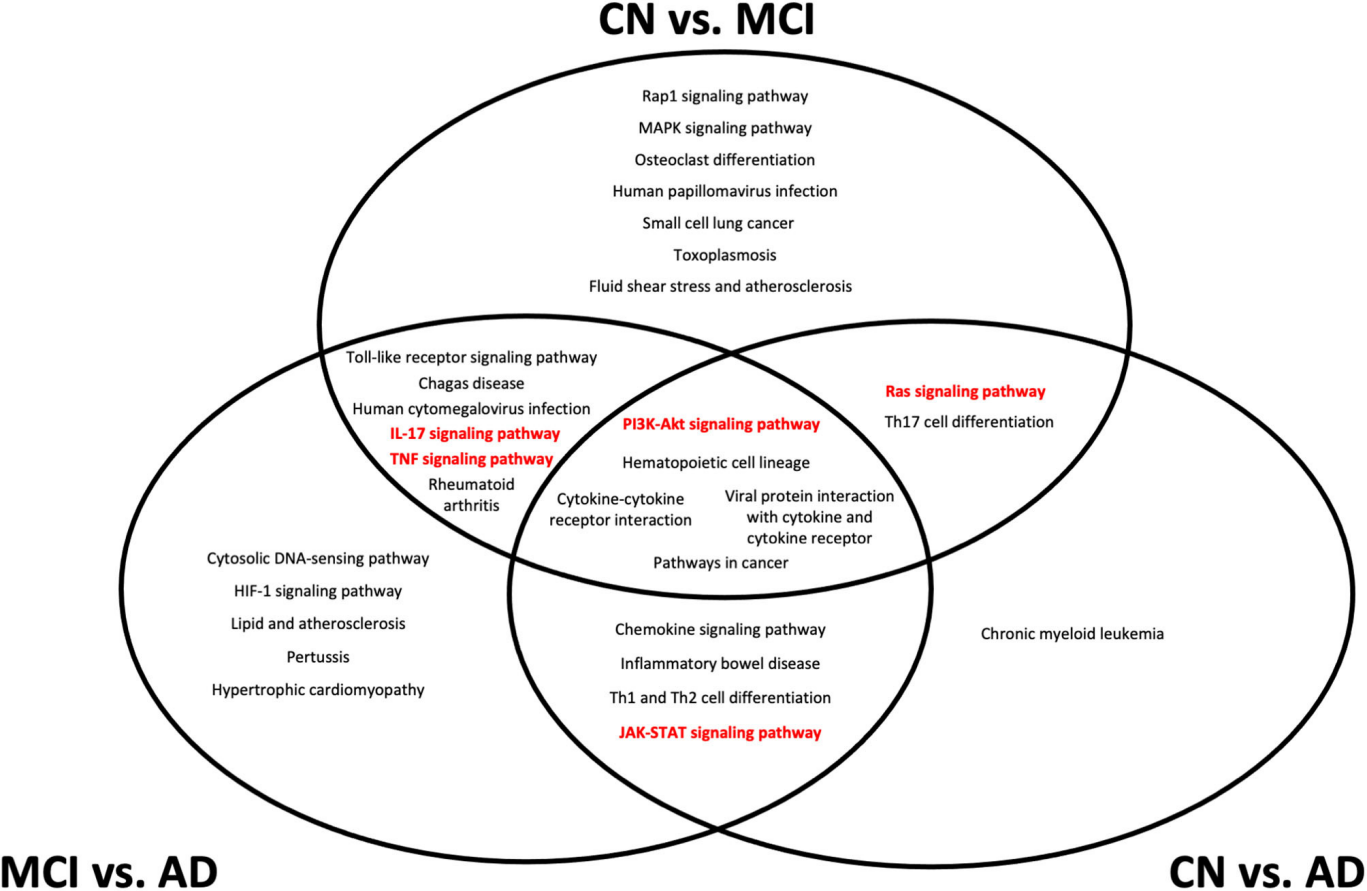
Top 20 Kyoto Encyclopedia of Genes and Genomes (KEGG) pathways from gene set enrichment analysis of differentially expressed genes from each pairwise comparison. All pathways shown have an adjusted *p* < 0.05 from gene set enrichment analysis. Pathways that are considered as potential biomarker pathways, specifically the PI3K-Akt, IL-17, JAK-STAT, TNF, and Ras signaling pathways, are shown in red. CN, cognitively normal; MCI, mild cognitive impairment; AD, Alzheimer's Disease.

In our analysis of the gene annotations of each set of genes obtained from pairwise Boruta analyses, we were interested in signaling pathways that were found in the intersection between the three pairwise comparisons. These overlapping regions indicate gene annotations that could be implicated during transition across CN, MCI, and AD development, and could present potential therapeutic targets. From the KEGG pathway annotations, we identified five such pathways, namely the PI3K-Akt, IL-17, JAK-STAT, TNF, and Ras signaling pathways (Figure 4).

The location of each pathway in the Venn diagram in Figure 4 indicates the stage at which the pathway is implicated in the transition between conditions. Pathways at the center of the Venn diagram, where all three pairwise comparisons overlap, likely undergo expression changes at the transcriptomic level throughout CN, MCI, and AD development. Pathways that overlap between CN vs. MCI and CN vs. AD are likely to undergo changes during MCI, which are retained into AD, while pathways that overlap between CN vs. MCI and MCI vs. AD likely undergo changes that are more detectable during MCI but not necessarily AD. Pathways that overlap between CN vs. AD and MCI vs. AD indicate changes that are likely to be more representative of AD, rather than MCI.

## Discussion

Visualization of the blood gene expression profile using UMAP did not show distinctive clusters of AD, MCI and CN, illustrating the heterogeneous nature of AD. The visualization under UMAP also demonstrated that the underlying structure of detecting biomarkers in AD could not be resolved easily even when using non-linear approaches. Therefore, conventional classification approaches will likely be ineffective and a robust machine learning algorithm will be required for a more accurate biomarker identification. In this regard, RF was chosen for its versatile applications in classification and regression, and Boruta was employed to further increase robustness. In this study, we employed a machine learning pipeline for the identification of AD-related genes, which in turn highlighted five signaling pathways that could potentially be used for discriminating between stages of AD progression.

We recognize that the ambiguity of a patient's true condition, under the MCI classification, may influence the feature selection as not all MCI patients evolve to AD, and some individuals can even revert back to CN. This suggests that the disease progression from MCI to AD is non-linear, as previously suggested in literature (34). The clinical diagnosis of MCI, upon which our feature selection algorithm depends, is also challenging due to the lack of clear distinguishing symptoms. In addition, the worsening of MCI may develop into other forms of dementia besides AD. Nevertheless, the association between MCI and AD development provides motivation for studying both of them in conjunction. By considering MCI and AD as different conditions, we aim to identify not only potential blood biomarkers for AD diagnosis, but also biomarkers for MCI diagnosis, with the goal to provide early intervention for AD by detection of MCI subjects before they progress into AD. Early detection of MCI subjects and treatment may alleviate their symptoms in the early stages and reduce the probability of MCI patients progressing into AD.

The genes identified in this study are able to classify subjects as belonging to either CN, MCI, or AD groups, with greater than random accuracy, thus supporting their potential application in biomarker panels for diagnostic purposes in clinical settings. However, the classification accuracies observed in this study also suggest that MCI and AD are highly complex and heterogeneous. This could have been a result of the gene expression landscape between healthy and diseased groups being relatively similar overall (35, 36). To reduce variability resulting from class imbalance in our data during feature selection and evaluation, we performed SMOTE on both our training and testing data, and employed several iterations of Boruta, with majority voting to reduce the likelihood of false positives being included in our final set of DEGs. Further validation and investigation of these DEGs as clinically relevant biomarkers for MCI and AD will ideally be conducted in the future using a much larger dataset (than the current NNI data used in this study), that is also supported by common AD biomarkers such as amyloid and tau.

Despite the discovery cohort being relatively small, our classification models using DEGs identified from NNI data also classified subjects with greater than random accuracy when validated on external ADNI data, with comparable prediction accuracies across all three pairwise comparisons of CN vs. MCI, CN vs. AD, and MCI vs. AD. Therefore, our DEGs can be generalized to different cohorts of subjects from varying geographical regions and data collection methods.

The five pathways (PI3K-Akt, IL-17, JAK-STAT, TNF, and Ras signaling) identified from gene enrichment analysis of the DEGs represent potential changes in biochemical signaling over different stages of MCI and AD onset and development. It is likely that all of the five pathways undergo some dysregulation during the course of MCI development and AD progression. The finding of inflammatory pathways in the differential gene expression analysis is not particularly surprising, given that the initial set of NNI genes were also associated with several inflammatory processes. Despite this, the localization of the different pathways to different regions of the Venn diagram in Figure 4 indicates that at different stages of disease progression, different pathways could experience more detectable transcriptomic changes. This has applications in diagnostic practice and personalized medicine–although MCI is associated with increased systemic inflammation in general (37), greater dysregulation of specific pathways could provide indications of disease stages in each patient, and provide potential targets for that disease stage.

Additional challenges remain to be addressed regarding blood transcriptomes as biomarkers for disease diagnosis. Relating the dynamics of gene expression in the brain to blood can be confounded by changes in RNA availability rather than specific pathway dysregulation, such as the possibility that extensive neuronal death in highly inflammatory environments could lead to less detectable RNA changes at later AD stages (38). Therefore, it would be prudent to consider if the observed transcriptional changes detected were indeed associated with disease progression.

Each of the five pathways identified in this study has individually been implicated in cognitive impairment, particularly when they are altered in the central nervous system, even in neurological conditions not directly linked to AD (39–43). Should their expression levels in blood aid in identifying individuals with cognitive impairment accurately, dysregulation of these pathways would be possible biomarkers for MCI. An association between brain and blood levels of their expression, with corroboration from behavioral studies, would further support the utility of these pathways as tools for assessing patients' risks of developing MCI and AD onset, providing opportunities for therapeutic intervention.

The five inflammatory pathways observed from gene enrichment have each been associated with AD, albeit usually in the context of pathway dysregulation in the brain. Of note, IL-17A demonstrates a strong correlation with the pathogenesis of AD, and evidence of elevated IL-17A was observed in AD patients (44). Studies performed on rodents with Aβ-induced neurodegeneration demonstrated improved memory function after treatment with IL-17 antibodies (45). This provides strong evidence that the inhibition of IL-17 reduces the degenerative effects of Aβ in the glial cells, preventing further complications in AD patients.

Another pathway that holds potential in the treatment of AD is the TNF signaling pathway. Numerous studies have described the elevation of TNF-α in AD patients (46, 47) and the intervention of this pathway has been shown to alleviate brain pathology in rodent models (48). Additionally, the role of the TNF signaling pathway is supported by a recent genome-wide association study, which suggests that regulation of this pathway and its interactions with other signaling pathways could be implicated in AD development and neuropathology (49). Several drugs have been proposed to target different parts of the TNF signaling pathway and there is some reported clinical evidence suggesting that intervention of this pathway lowers AD neuropathology (50). Currently there are a few FDA approved drugs that target the IL-17A signaling and TNF signaling pathways. A few drug examples include Secukinumab, an antibody that selectively binds and neutralizes IL-17A, and Etanercept, a biological antagonist to TNF-α, that has been shown in a pilot study to improve cognitive function (51). This information may be applicable in the treatment of MCI patients, if early detection and diagnosis is conducted through routine blood tests, to prevent the potential development of AD.

Additionally, the JAK-STAT signaling and PI3K-Akt signaling pathways were found to be differentially expressed between AD, MCI and CN, serving as potential grounds for future research or drug targets. Although the mechanism behind how these pathways lead to development of AD is unclear, past investigations have found correlations between altered activity of these pathways and development of AD. The JAK-STAT signaling pathway was found to be activated in reactive astrocytes present in rodent models (52), although the mechanism of how JAK-STAT is involved in AD has not been studied extensively. The PI3K-Akt signaling pathways were found to be inhibited by Aβ, leading to neuronal death (53), but the mechanism of how this occurs is still not well understood. The involvement of these two pathways in AD pathophysiology could be explored in further research, with the possibility of them being therapeutic targets.

Ras/ERK signaling and its associated MAPK signaling pathway have also been investigated widely in contexts of disease due to their diverse regulatory roles in processes such as cell survival, migration, proliferation, and differentiation. In AD, ERK proteins are hypothesized to play a role in mediating Tau hyperphosphorylation and β-secretase expression which influences Aβ aggregation (54). Ras/ERK signaling has also been shown to be activated by Aβ, with the resultant aberrant signaling leading to neurodegeneration in AD (41). Therefore, early detection and targeted treatment for Ras signaling dysregulation could provide a means for preventing prolific Aβ aggregation and neurodegeneration in early AD.

Differential expression in these five pathways indicates that these pathways are dysregulated at different stages of disease development over MCI and AD, although it should be noted that the complex relationship between blood and brain expression makes interpretation of blood gene expression difficult, specifically in understanding how differential expression in blood is indicative of transcriptional patterns in the brain. The transcriptional signature of some gene modules in the brain, particularly those with roles in basic cellular processes such as gene expression regulation and infection, have been observed to be preserved in the blood of healthy subjects (55). In AD patients, especially at advanced stages of disease, strong blood-brain correlation for transcription has been observed, including brain-specific genes and inflammation-associated genes (56). However, blood-brain transcriptional correlation in MCI subjects remains to be established. While further investigation is needed before it can be concluded if these patterns of dysregulation definitively reflect neurophysiological changes in the brain, the findings of past human and animal studies suggest that the blood and brain transcriptome are correlated for some pathways, including inflammatory genes such as those studied here, especially in neurodegeneration (57).

Another challenge in identifying blood biomarkers of disease relates to population heterogeneity. Highly generalisable biomarkers are rarely available, due to heterogeneity between individuals, even within the healthy control population–the blood transcriptome is highly dynamic and inflammatory processes may vary in response to age, underlying disease, viral infection, or even seasonal changes (16).

This not only presents an opportunity for personalized medicine, but also highlights its importance in MCI and AD diagnosis. Since the expression of these inflammatory pathways is dynamic and highly variable even in healthy subjects, there is a risk of false positives in MCI and AD diagnosis if one only considers differences in expression levels of genes in the five pathways between individuals. Consequently, in evaluating MCI and AD risk of an individual, it may be beneficial to track longitudinal changes in expression of the five pathways for each subject, rather than making a diagnosis based on data from a single time point.

There are several previously published studies that aim to identify blood biomarkers of AD using blood gene expression data (14, 34–36, 58–60). A number of these studies employed publicly available data from AddNeuroMed for biomarker discovery and evaluation, although the different methods for feature selection and classification employed for each study resulted in varying differentially expressed features between AD and CN subjects (14, 34, 35, 59, 60). To overcome variations in individual differentially expressed genes, researchers may employ pathway-based classification models instead (14, 36), which we also include in our investigation. Through pathway analysis, there is more consensus between studies–for instance, the JAK-STAT pathway we identify in this investigation was also mentioned as a pathway of interest by Li et al. (35). Here, our study differs from previously published work in that we focus on biomarkers of MCI as indicators of AD risk, and on the pathways that are implicated across the development of MCI and AD, rather than just differential expression at each stage compared to CN.

The findings of this study provide potential blood biomarker genes and pathways for assessing MCI and AD risk in individuals. However, the complex molecular landscape of MCI and AD presents opportunities for further research, with the aim of making definitive diagnoses based on RNA extracted from blood. For instance, the relationship between blood RNA levels and RNA and protein levels in the brain could be further explored to determine how the differences observed in blood between conditions are linked to physiological changes in the brain during AD progression. Another aspect that could be considered is how the biomarkers identified in this study compare against blood biomarkers for other diseases, especially inflammatory conditions.

## Conclusions

A machine learning pipeline employing Boruta effectively identifies DEGs in AD, which can classify subjects as either MCI or AD patients. These genes are enriched in five pathways–the PI3K-Akt, IL-17, JAK-STAT, TNF, and Ras signaling pathways–which are possibly dysregulated in MCI and AD. These pathways are also potential biomarkers that classify MCI and AD patients with reasonable accuracy. Our study demonstrates the potential to make use of these discovered biomarkers for early diagnosis of MCI and AD patients through routine blood testing, thereby providing biological insights toward early intervention through targeted drug treatment development for preventing deterioration into AD. These biomarker pathways may also provide targets for disease monitoring over time as an approach for personalized medicine as their transcriptional patterns are altered as MCI develops into AD.

## Data Availability Statement

ADNI datasets are publicly available (ADNI, http://adni.loni.usc.edu/). The NNI data that support the findings of this study are available from the corresponding authors, EG and K-HC, upon request.

## Author Contributions

SLO did the data analysis, prepared figures, and wrote the manuscript. MKZ assisted with data analysis, figure preparation, and manuscript writing. EWMC, GA, and CHC together did sample preparation and primer optimisations, designed Fluidigm chips, did the assays and data validation, and were involved in data analysis and drafting of the manuscript. KPN and NK collected and prepared patients samples, and carried out clinical evaluation of psychological and cognitive performance. EG initiated and directed the study. K-HC directed the bioinformatics study and analysis. All authors contributed to the article and approved the submitted version.

## Funding

This study was supported by the Ministry of Education (MOE) Tier 3 grant (MOE2017-T3-1-002) to EG.

## Conflict of Interest

The authors declare that the research was conducted in the absence of any commercial or financial relationships that could be construed as a potential conflict of interest.

## Publisher's Note

All claims expressed in this article are solely those of the authors and do not necessarily represent those of their affiliated organizations, or those of the publisher, the editors and the reviewers. Any product that may be evaluated in this article, or claim that may be made by its manufacturer, is not guaranteed or endorsed by the publisher.
