# Supplementary material for: Alzheimer's Disease Blood Biomarkers Associated With Neuroinflammation as Therapeutic Targets for Early Personalized Intervention
Source: Front Digit Health. 2022 Jul 11;4:875895. doi: 10.3389/fdgth.2022.875895 (PMC9309434; doi:10.3389/fdgth.2022.875895)
Supplement: Supplementary file 1 [file Table_1.docx]

# Supplementary Material

**Supplementary Table 1: Target genes studied for NNI data**

| **Target No.** | **Gene** | **Gene Name** | **Function** | **Reference** | **Remarks** |
| --- | --- | --- | --- | --- | --- |
| HK1 | *GAPDH* | Glyceraldehyde-3- Phosphate  Dehydrogenase | Enzyme involved in glucose metabolism | (Karch et al., 2012) |  |
| HK2 | *RP2* | ARL3 GTPase activating protein | Involved in trafficking between the Golgi and the ciliary membrane | (Wright et al., 2011) |  |
| HK3 | *TBP* | TATA-Box Binding  Protein | Transcription factor that binds to TATA box site | (Akhtar & Veenstra, 2011) |  |
| HK4 | *CYC1* | Cytochrome C1 | Complex involved in mitochondrial respiration | (Penna et al., 2011) |  |
| HK5 | *UBC* | Ubiquitin C | Maintenance of cellular homeostasis | (Park & Ryu, 2014) |  |
| Target 1 | *ADIPOQ* | Adiponectin | Adipokine involved in the control of fat metabolism, with direct anti-diabetic, anti-atherogenic and anti-inflammatory activities. | (O’Bryant et al., 2011) |  |
| Target 2 | *ADM* | Adrenomedullin | Involved in vasodilation, regulation of hormone secretion, promotion of angiogenesis, and antimicrobial activity. | (Buerger et al., 2010) |  |
| Target 3 | *CLU* | Clusterin | Involved in synapse turnover, apoptosis, cytoprotection at fluid- tissue boundaries and regulation of complement-mediated  membrane attack complex. | (Karch et al., 2012) |  |
| Target 4 | *ARG1* | Arginase 1 | Critical regulator of innate and adaptive immune responses. | (Das & Chinnathambi, 2019) |  |
| Target 5 | *BECN1* | Beclin 1 | Regulates autophagy, and degradation of Aβ plaques. | (Swaminathan et al., 2016) |  |
| Target 6 | *CCL15* | C-C Motif Chemokine Ligand 15 | Chemotactic factor that attracts T-cells and monocytes, but not neutrophils, eosinophils, or B-cells. | (Ray et al., 2007) | Filtered out before analysis |
| Target 7 | *CCL2* | C-C Motif Chemokine Ligand 2 | Chemokine Involved in immunoregulatory and inflammatory processes. | (Ray et al., 2007) |  |
| Target 8 | *CCL22* | C-C Motif Chemokine Ligand 22 | Displays chemotactic activity for monocytes, dendritic cells, natural killer cells and for chronically activated T lymphocytes. | (Ray et al., 2007) |  |
| Target 9 | *CCL5* | C-C Motif Chemokine Ligand 5 | Chemoattractant for blood monocytes, memory T helper cells and eosinophils and causes the release of histamine. | (Ray et al., 2007) |  |
| Target 10 | *CCL4* | C-C Motif Chemokine Ligand 4 | Monokine with inflammatory and chemokinetic properties. | (Ray et al., 2007) |  |
| Target 11 | *CCL17* | C-C Motif Chemokine Ligand 17 | Chemokine plays important roles in T cell development in thymus as well as in trafficking and activation of mature T cells. | (Ray et al., 2007) |  |
| Target 12 | *THBD* | Thrombomodulin | Serves as a cofactor for thrombin and reduces blood coagulation by converting thrombin to an anticoagulant enzyme. | (O’Bryant et al., 2011) |  |
| Target 13 | *ACE* | Angiotensin I Converting Enzyme | Converts angiotensin I to angiotensin II which results in an increase of the vasoconstrictor activity of angiotensin. Also able to inactivate bradykinin, a potent vasodilator. | (O’Bryant et al., 2011) |  |
| Target 14 | *PECAM1* | Platelet And Endothelial Cell Adhesion Molecule 1 | Member of the immunoglobulin superfamily and is likely involved in leukocyte migration, angiogenesis, and integrin activation. | (Kalinowska & Losy, 2006) |  |
| Target 15 | *CKM* | Creatine Kinase, M-Type | Cytoplasmic enzyme involved in energy homeostasis and is an important serum marker for myocardial infarction. | (Spindler et al., 2002) |  |
| Target 16 | *CSF1* | Colony Stimulating Factor 1 | Cytokine that plays an essential role in the regulation of survival, proliferation and differentiation of macrophages and monocytes. Promotes the release of proinflammatory chemokines. | (Ray et al., 2007) |  |
| Target 17 | *CXCL10* | C-X-C Motif Chemokine Ligand 10 | Binds to CXCR3 results in pleiotropic effects, including stimulation of monocytes, natural killer and T-cell migration, and modulation of adhesion molecule expression. | (Brosseron et al., 2014) |  |
| Target 18 | *CXCL12* | C-X-C Motif Chemokine Ligand 12 | a role in many diverse cellular functions, including embryogenesis, immune surveillance, inflammation response, tissue homeostasis, and tumor growth and metastasis | (Ray et al., 2007) |  |
| Target 19 | *EGF* | Epidermal Growth Factor | Potent mitogenic factor that plays an important role in the growth, proliferation and differentiation of numerous cell types. | (Ray et al., 2007) |  |
| Target 20 | *FASLG* | Fas Ligand | FAS/FASLG signaling pathway is essential for immune system regulation and cytotoxic T lymphocyte induced cell death. | (O’Bryant et al., 2011) |  |
| Target 21 | *RETNLB* | Resistin Like Beta | May contribute to hypoxic induced vascular remodeling processes or hypoxia related inflammation. | (Bednarska-Makaruk et al., 2017) |  |
| Target 22 | *IFNB1* | Interferon Beta 1 | Interferon involved in cell differentiation and defense against viral infection. | (Zheng et al., 2016) |  |
| Target 23 | *IGF1* | Insulin Like Growth Factor 1 | Positive regulation of protein phosphorylation, synapse maturation, activation of the PI3K-AKT/PKB and Ras-MAPK  pathways. | (Ray et al., 2007) | Filtered out before analysis |
| Target 24 | *IGFBP6* | Insulin Like Growth Factor Binding Protein 6 | Stimulate the growth promoting effects of the IGFs and alter the interaction of IGFs with their cell surface receptors. | (Ray et al., 2007) |  |
| Target 25 | *CHUK* | Component Of Inhibitor Of Nuclear Factor Kappa B Kinase Complex | Cytokine-activated protein complex that is an inhibitor of NFKB complex. | (Van Eldik et al., 2016) |  |
| Target 26 | *IKBKB* | Inhibitor Of Nuclear Factor Kappa B Kinase Subunit Beta | Regulatory factor that is activated by inflammatory cytokines, infection, DNA damages or other cellular stresses. | (Van Eldik et al., 2016) |  |
| Target 27 | *IKBKG* | Inhibitor Of Nuclear Factor Kappa B Kinase Regulatory Subunit  Gamma | Activates NFKB resulting in activation of genes involved in inflammation, immunity, cell survival, and other pathways. | (Van Eldik et al., 2016) |  |
| Target 28 | *IL10* | Interleukin 10 | Down-regulates the expression of Th1 cytokines and shows anti-inflammatory functions. Also enhances B cell survival, proliferation, and antibody production. This cytokine can block NFKB activity. | (Ray et al., 2007) |  |
| Target 29 | *IL11* | Interleukin 11 | Support the proliferation of hematopoietic stem cells and megakaryocyte progenitor cells. | (Ray et al., 2007) | Filtered out before analysis |
| Target 30 | *IL12A* | Interleukin 12A | Cytokine required for the T-cell-independent induction of interferon (IFN)-gamma, and is important for the differentiation of both Th1 and Th2 cells. | (Lin et al., 2019) |  |
| Target 31 | *IL15* | Interleukin 15 | Regulates T and natural killer cell activation and proliferation. | (Ray et al., 2007) |  |
| Target 32 | *IL17A* | Interleukin 17A | Proinflammatory cytokine produced by activated T cells. Regulates the activities of NF-kappaB and MAPK and also stimulates the expression of IL6 and (PTGS2/COX-2), as well as  enhance the production of nitric oxide (NO). | (Yang et al., 2017) |  |
| Target 33 | *IL18* | Interleukin 18 | Proinflammatory cytokine that stimulates inflammatory responses via production of interferon gamma from T-helper type I cells. | (Brosseron et al., 2014) |  |
| Target 34 | *IL1A* | Interleukin 1 Alpha | IL-1 stimulates thymocyte proliferation, B-cell maturation and proliferation, and fibroblast growth factor activity. IL-1 proteins are involved in the inflammatory response. | (Ray et al., 2007) |  |
| Target 35 | *IL1B* | Interleukin 1 Beta | Induces prostaglandin synthesis, neutrophil activation, T-cell activation and cytokine production, B-cell activation and  antibody production, and fibroblast proliferation and collagen production. | (Ray et al., 2007) |  |
| Target 36 | *IL23* | Interleukin 23 | Cytokine which functions in innate and adaptive immunity and  promotes production of proinflammatory cytokine. | (Liu et al., 2014) |  |
| Target 37 | *IL2RA* | Interleukin 2 Receptor Subunit Alpha | Receptor involved in the regulation of immune tolerance by controlling regulatory T cells (TREGs) activity. | (Ray et al., 2007) |  |
| Target 38 | *IL6* | Interleukin 6 | Cytokine with a wide variety of biological function such as acute phase response, differentiation of B-cells into Ig-secreting cells, differentiation of lymphocyte and monocyte. | (Ray et al., 2007) |  |
| Target 39 | *IL7* | Interleukin 7 | Hematopoietic growth factor capable of stimulating the proliferation of lymphoid progenitors. It is important for proliferation during certain stages of B-cell maturation. | (Ray et al., 2007) |  |
| Target 40 | *LPA* | Lipoprotein(A) | Involved in inhibition of activity of tissue-type plasminogen activator I. | (O’Bryant et al., 2011) |  |
| Target 41 | *LIF* | LIF Interleukin 6 Family Cytokine | Involved in the induction of hematopoietic differentiation, neuronal cell differentiation and may also have a role in immune tolerance. | (Brosseron et al., 2014) | Filtered out before analysis |
| Target 42 | *NTF3* | Neurotrophin 3 | Promote the survival of visceral and proprioceptive sensory neurons. | (Ray et al., 2007) |  |
| Target 43 | *NTF4* | Neurotrophin 4 | Target-derived survival factor for peripheral sensory sympathetic neurons. | (Ray et al., 2007) |  |
| Target 44 | *SELP* | Selectin P | Protein redistributes to the plasma membrane during platelet activation and mediates the interaction of activated endothelial cells or platelets with leukocytes. | (Grammas, 2011) |  |
| Target 45 | *PTX3* | Pentraxin 3 | Promotes fibrocyte differentiation and is involved in regulating inflammation and complement activation. Also plays a role in angiogenesis and tissue remodelling. The protein serves as a biomarker for several inflammatory conditions. | (Slusher et al., 2019) |  |
| Target 46 | *KITLG* | KIT Ligand | Pleiotropic factor that is involved in neural cell development  and hematopoiesis. | (Ray et al., 2007) |  |
| Target 47 | *THPO* | Thrombopoietin | Humoral growth factor that is necessary for megakaryocyte proliferation and maturation, as well as for thrombopoiesis. | (Ray et al., 2007) |  |
| Target 48 | *TNFRSF1B* | TNF Receptor Superfamily Member 1B | Mediates the recruitment of anti-apoptotic proteins. | (Ray et al., 2007) |  |
| Target 49 | *TNF* | Tumor Necrosis Factor | Cytokine is mainly secreted by macrophages and is involved in  the regulation of cell proliferation, differentiation, apoptosis, lipid metabolism, and coagulation. | (Julian et al., 2018) |  |
| Target 50 | *TNFSF10* | TNF Superfamily Member 10 | Protein preferentially induces apoptosis in transformed and tumor cells, but does not appear to kill normal cells. | (Brosseron et al., 2014) |  |
| Target 51 | *VEGFA* | Vascular Endothelial Growth Factor A | Induces proliferation and migration of vascular endothelial cells, and is essential for both physiological and pathological angiogenesis. | (Brosseron et al., 2014) |  |
| Target 52 | *VWF* | Von Willebrand Factor | Function in the adhesion of platelets to sites of vascular injury and the transport of various proteins in the blood. | (Grammas, 2011) |  |
| Target 53 | *B2M* | Beta-2-Microglobulin | Component of the class I major histocompatibility complex (MHC). Involved in the presentation of peptide antigens to the  immune system. | (O’Bryant et al., 2011) |  |
| Target 54 | *NOS2* | Nitric Oxide Synthase 2 | Biologic mediator in several processes, including neurotransmission and antimicrobial and antitumoral activities. | (Van Eldik et al., 2016) |  |
| Target 55 | *CD163* | CD163 Molecule | Involved in endocytosis of hemoglobin/haptoglobin complexes by macrophages, and may also function as an innate immune sensor for bacteria and local inflammation | (Pey et al., 2014) |  |
| Target 56 | *CHI3L1* | Chitinase 3 Like 1 | Plays a role in T-helper cell type 2 (Th2) inflammatory response and IL-13-induced inflammation, inflammatory cell apoptosis, dendritic cell accumulation and M2 macrophage differentiation. | (Craig-Schapiro et al., 2010) |  |
| Target 57 | *TLR4* | Toll Like Receptor 4 | Key role in the innate immune system. | (Zhang et al., 2012) |  |
| Target 58 | *OSM* | Oncostatin M | Secreted cytokine and growth regulator that inhibits the proliferation | (Ray et al., 2007) |  |
| Target 59 | *OCLN* | Occludin | Forms part of the tight junctions between brain endothelial cells that control blood-brain barrier (BBB) function. | (Bai et al., 2014) |  |
| Target 60 | *ITGB3* | Integrin Subunit Beta 3 | Clot formation and promote wound healing. | (Bai et al., 2014) |  |
| Target 61 | *CA4* | Carbonic Anhydrase 4 | Critical role in pH regulation and long-term synaptic transformation. | (Bai et al., 2014) |  |
| Target 62 | *CCR2* | C-C Motif Chemokine Receptor 2 | Chemokine favoring monocyte/ macrophage migration through the BBB. | (Guedes et al., 2018) |  |
| Target 63 | *CXCR3* | C-X-C Motif Chemokine Receptor 3 | Leukocyte traffic and most notably integrin activation. | (Guedes et al., 2018) |  |
| Target 64 | *CX3CL1* | C-X3-C Motif Chemokine Ligand 1 | Anti-inflammatory cytokine is expressed on neurons. | (Ray et al., 2007) |  |
| Target 65 | *IL6R* | Interleukin 6 Receptor | Immune and inflammatory response | (Ray et al., 2007) |  |
| Target 66 | *TOMM40* | Translocase Of Outer Mitochondrial  Membrane 40 | Essential for import of protein precursors into mitochondria | (T.-S. Lee et al., 2012) |  |
| Target 67 | *DNMT3A* | DNA Methyltransferase 3 Alpha | Important role in the development of embryogenesis and the generation of aberrant methylation late-onset AD (LOAD) | (Ling et al., 2016) |  |
| Target 68 | *ASXL1* | ASXL Transcriptional Regulator 1 | Role in chromatin remodeling, regulates the expression HOX genes, methylation of promotor regions | (Bouzid et al., 2021) |  |
| Target 69 | *BCOR* | BCL6 Corepressor | Via repression of TFAP2A acts as a negative regulator of osteo- dentiogenic capacity in adult stem cells; the function implies inhibition of methylation on histone H3 'Lys-4' and 'Lys-36' | (Baron et al., 2015) |  |
| Target 70 | *BCORL1* | BCL6 Corepressor Like 1 | Encoded protein can interact with several different class II histone deacetylases to repress transcription. | (Rangaraju et al., 2018) |  |
| Target 71 | *SF3B1* | Splicing Factor 3b Subunit 1 | Encodes the U2 small nuclear riboprotein complex (snRNP) responsible for 3′ branch site recognition and is the most frequently mutated splicing factor gene. | (Wong, 2013) |  |
| Target 72 | *SRSF2* | Serine And Arginine Rich Splicing Factor 2 | Splicing factors that interacts with the U2 and U1 components of the spliceosome. | (Qian & Liu, 2014) |  |
| Target 73 | *U2AF1* | U2 Small Nuclear RNA Auxiliary Factor 1 | U2 spliceosome that is responsible for recognizing the AG splice  acceptor dinucleotide at the 3′ end of introns. | (Rybak-Wolf & Plass, 2021) |  |
| Target 74 | *JAK2* | Janus Kinase 2 | Protein is especially important for controlling the production of blood cells from hematopoietic stem cells. | (Chiba et al., 2009) |  |
| Target 75 | *LEPR* | Leptin Receptor | Regulate food intake and energy homeostasis. Inducers of vascular epithelial cell growth and angiogenesis. | (Bednarska-Makaruk et al., 2017) |  |
| Target 76 | *ADAM10* | ADAM Metallopeptidase Domain 10 | α-secretases involved in APP cleavage. | (Wongchitrat et al., 2019) |  |
| Target 77 | *RNF213* | Ring Finger Protein 213 | Plays a role in the proper development of blood vessels. | (Bai et al., 2014) |  |
| Target 78 | *CXCR4* | C-X-C Motif Chemokine Receptor 4 | Regulation of neuroinflammatory responses. | (Guedes et al., 2018) |  |
| Target 79 | *CXCR2* | C-X-C Motif Chemokine Receptor 2 | Regulation of neuroinflammatory responses. | (Guedes et al., 2018) |  |
| Target 80 | *CCR5* | C-C Motif Chemokine Receptor 5 (Gene/Pseudogene) | Play a role in granulocyte lineage proliferation and differentiation | (Guedes et al., 2018) |  |
| Target 81 | *CBL* | Cbl Proto-Oncogene | Participates in proteasome degradation and signal transduction in hematopoietic cells. | (Nho et al., 2017) |  |
| Target 82 | *GNB1* | G Protein Subunit Beta 1 | G proteins which integrate signals between receptors and  effector proteins. | (Manavalan et al., 2013) |  |
| Target 83 | *STAT3* | Signal Transducer And Activator Of Transcription 3 | Role in differentiation of the TH17 helper T cells, cell growth and apoptosis. | (Chiba et al., 2009) |  |
| Target 84 | *NFKB1* | Nuclear Factor Kappa B  Subunit 1 | Stimulates the expression of genes involved in a wide variety of  biological functions. | (Ascolani et al., 2012) |  |
| Target 85 | *IL32* | Interleukin 32 | Induces the production of TNFalpha from macrophage cell. | (Yun et al., 2015) |  |
| Target 86 | *SOD1* | Superoxide Dismutase 1 | Enzyme involved in production of charged oxygen molecules called superoxide radicals. | (Gatta et al., 2009) |  |
| Target 87 | *BAX* | BCL2 Associated X, Apoptosis Regulator | Protein forms a heterodimer with BCL2 and functions as an apoptotic activator. | (Gatta et al., 2009) |  |
| Target 88 | *NOTCH1* | Notch Receptor 1 | Key role in neural development. | (Kofler et al., 2015) |  |
| Target 89 | *S100A8* | S100 Calcium Binding Protein A8 | Induce neutrophil chemotaxis and adhesion. | (Cristóvão & Gomes, 2019) |  |
| Target 90 | *BIN1* | Bridging Integrator 1 | Involved in the regulation of intracellular vesicles sorting, modulation of BACE1 trafficking and the control of amyloid-  beta production. | (Karch et al., 2012) |  |
| Target 91 | *PICALM* | Phosphatidylinositol Binding Clathrin Assembly Protein | Cytoplasmic adapter protein that plays a critical role in clathrin- mediated endocytosis for internalization of cell receptors, synaptic transmission or removal of apoptotic cells. | (Karch et al., 2012) |  |
| Target 92 | *SERPINA3* | Serpin Family A Member 3 | Plasma protease inhibitor and member of the serine protease inhibitor class | (Kamboh et al., 2006) | Filtered out before analysis |
| Target 93 | *ANGPT2* | Angiopoietin 2 | Disrupts the vascular remodeling ability of ANGPT1 and may induce endothelial cell apoptosis, cell migration and proliferation, thus serving as a permissive angiogenic signal. | (Ray et al., 2007) | Filtered out before analysis |
| Target 94 | *NPPA* | Natriuretic Peptide A | Control of extracellular fluid volume and electrolyte homeostasis. | (Mahinrad et al., 2018) |  |
| Target 95 | *BDNF* | Brain Derived Neurotrophic Factor | Promotes the survival and differentiation of selected neuronal populations of the peripheral and central nervous systems | (Ray et al., 2007) |  |
| Target 96 | *NPPB* | Natriuretic Peptide B | Natriuresis, diuresis, vasorelaxation, inhibition of renin and aldosterone secretion, and a key role in cardiovascular  homeostasis. | (Mahinrad et al., 2018) |  |
| Target 97 | *CCL18* | C-C Motif Chemokine Ligand 18 | Involved in immunoregulatory and inflammatory process | (Ray et al., 2007) |  |
| Target 98 | *CCL27* | C-C Motif Chemokine Ligand 27 | Play a role in mediating homing of lymphocytes to cutaneous sites | (Ray et al., 2007) |  |
| Target 99 | *CCL7* | C-C Motif Chemokine Ligand 7 | Secreted chemokine which attracts macrophages during inflammation and metastasis | (Ray et al., 2007) |  |
| Target 100 | *CCL13* | C-C Motif Chemokine Ligand 13 | Involved in the recruitment of monocytes into the arterial wall during the disease process of atherosclerosis. | (Ray et al., 2007) |  |
| Target 101 | *CCL3* | C-C Motif Chemokine Ligand 3 | Monokine with inflammatory and chemokinetic properties | (Ray et al., 2007) |  |
| Target 102 | *F3* | Coagulation Factor III, Tissue Factor | Initiates blood coagulation by forming a complex with circulating factor VII or VIIa. | (Bots et al., 1998) |  |
| Target  103 | *CD40LG* | CD40 Ligand | It regulates B cell function by engaging CD40 on the B cell  surface. | (Brosseron et al., 2014) |  |
| Target 104 | *SELE* | Selectin E | Responsible for the accumulation of blood leukocytes at sites of inflammation by mediating the adhesion of cells to the vascular lining. | (Grammas, 2011) | Filtered out before analysis |
| Target 105 | *PTGS1* | Prostaglandin-  Endoperoxide Synthase 1 | Encoded protein regulates angiogenesis in endothelial cells. | (Grammas, 2011) |  |
| Target 106 | *PTGS2* | Prostaglandin- Endoperoxide Synthase 2 | Responsible for the prostanoid biosynthesis involved in inflammation and mitogenesis. | (Grammas, 2011) |  |
| Target 107 | *CRP* | C-Reactive Protein | Involved in host defence function such as recognition of foreign pathogens and damaged cells. Protein levels in plasma increases during tissue injury, infection, or inflammatory stimuli. | (Grammas, 2011) |  |
| Target 108 | *CSF2* | Colony Stimulating Factor 2 | Cytokine that stimulates the growth and differentiation of hematopoietic precursor cell. | (Ray et al., 2007) |  |
| Target 109 | *CSF3* | Colony Stimulating Factor 3 | Cytokine involved in production, differentiation, and function of white blood cell populations. | (Ray et al., 2007) |  |
| Target 110 | *EDN1* | Endothelin 1 | Potent vasoconstrictor. | (Palmer et al., 2012) |  |
| Target 111 | *CXCL1* | C-X-C Motif Chemokine Ligand 1 | Growth factor that plays a role in inflammation and as a chemoattractant for neutrophils. | (Ray et al., 2007) |  |
| Target 112 | *CXCL9* | C-X-C Motif Chemokine Ligand 9 | Cytokine that affects the growth, movement, or activation state of cells that participate in immune and inflammatory response. | (Ray et al., 2007) |  |
| Target 113 | *CCL11* | C-C Motif Chemokine Ligand 11 | Promotes the accumulation of eosinophils, a prominent feature of allergic inflammatory reactions. | (Ray et al., 2007) |  |
| Target 114 | *FGF1* | Fibroblast Growth Factor 1 | Angiogenic factor involved in embryonic development, cell growth, morphogenesis, tissue repair, tumor growth and invasion. | (Brosseron et al., 2014) |  |
| Target 115 | *FGF2* | Fibroblast Growth Factor 2 | Growth factor can induce angiogenesis and involved in embryonic development and wound healing. | (Brosseron et al., 2014) |  |
| Target 116 | *GDNF* | Glial Cell Derived Neurotrophic Factor | Neurotrophic factor that promote the survival and differentiation of neurons in culture. | (Ray et al., 2007) |  |
| Target 117 | *IL6ST* | Interleukin 6 Signal Transducer | Regulate immune response, hematopoiesis, pain control and bone metabolism, embryonic development, survival of motor and sensory neurons. | (Haddick et al., 2017) |  |
| Target 118 | *HGF* | Hepatocyte Growth Factor | Role in angiogenesis, tumorogenesis, and tissue regeneration. | (Ray et al., 2007) |  |
| Target 119 | *TNNT2* | Troponin T2, Cardiac Type | Regulates muscle contraction in response to alterations in intracellular calcium ion concentration. | (Ray et al., 2007) |  |
| Target 120 | *ICAM1* | Intercellular Adhesion Molecule 1 | Role in cell proliferation, differentiation, motility, trafficking, apoptosis and tissue architecture. | (Ray et al., 2007) |  |
| Target 121 | *IFNA2* | Interferon Alpha 2 | Cytokine produced in response to viral infection. | (Brosseron et al., 2014) |  |
| Target 122 | *IFNG* | Interferon Gamma | Produced by lymphocytes and has important immunoregulatory functions. It is a potent activator of macrophages and has antiproliferative effects on transformed  cells. | (Ray et al., 2007) |  |
| Target 123 | *IL1RN* | Interleukin 1 Receptor Antagonist | Inhibits the activities of interleukin 1, alpha (IL1A) and interleukin 1, beta (IL1B), and modulates a variety of interleukin 1 related immune and inflammatory responses. | (Ray et al., 2007) |  |
| Target 124 | *IL1R1* | Interleukin 1 Receptor  Type 1 | Mediates interleukin-1-dependent activation of NF-kappa-B and MAPK pathways. | (Ray et al., 2007) |  |
| Target 125 | *IL1R2* | Interleukin 1 Receptor  Type 2 | Serves as a decoy receptor by competitive binding to IL1B and  preventing its binding to IL1R1 and modulates cellular responses. | (Ray et al., 2007) |  |
| Target 126 | *IL12B* | Interleukin 12B | Cytokine is expressed by activated macrophages that serve as an essential inducer of Th1 cells development. | (Ray et al., 2007) |  |
| Target 127 | *IL13* | Interleukin 13 | Involved in B-cell maturation and differentiation. Also, down- regulates macrophage activity and inhibits production of pro- inflammatory cytokines and chemokines. | (Ray et al., 2007) |  |
| Target  128 | *IL16* | Interleukin 16 | Functions as a chemoattractant and as a modulator of T cell  activation. | (Ray et al., 2007) |  |
| Target 129 | *IL2* | Interleukin 2 | Required for T-cell proliferation and regulation of the immune response. Can stimulate B-cells, monocytes, lymphocyte- activated killer cells, natural killer cells, and glioma cells. | (Ray et al., 2007) | Filtered out before analysis |
| Target  130 | *IL4* | Interleukin 4 | Involved in B-cell activation and autophagy in dendritic cells. | (Ray et al., 2007) |  |
| Target 131 | *IL5* | Interleukin 5 | Factor that induces terminal differentiation of late-developing B-cells to immunoglobulin secreting cells. | (Ray et al., 2007) |  |
| Target 132 | *CXCL8* | C-X-C Motif Chemokine Ligand 8 | Mediator of the inflammatory response and also a potent angiogenic factor. | (Ray et al., 2007) |  |
| Target 133 | *IL9* | Interleukin 9 | Supports IL-2 independent and IL-4 independent growth of helper T-cells. | (Ray et al., 2007) |  |
| Target 134 | *MIF* | Macrophage Migration Inhibitory Factor | Regulation of macrophage function in host defense through the suppression of anti-inflammatory effects of glucocorticoids. | (Ray et al., 2007) |  |
| Target 135 | *MMP2* | Matrix Metallopeptidase 2 | Enzyme capable of cleaving components of the extracellular matrix and molecules involved in signal transduction. | (Du et al., 2017) |  |
| Target 136 | *MMP9* | Matrix Metallopeptidase 9 | Involved in the breakdown of extracellular matrix in physiological processes, such as embryonic development,  reproduction and tissue remodeling. | (Turner & Sharp, 2016) |  |
| Target 137 | *MYD88* | MYD88 Innate Immune Signal Transduction Adaptor | Adapter protein involved in many signaling pathway to facilitate innate immune response. | (Van Eldik et al., 2016) |  |
| Target 138 | *NGF* | Nerve Growth Factor | Growth factor important for development and maintenance of sympathetic and sensory nervous systems. Also regulate neuronal proliferation, differentiation and survival. | (Ray et al., 2007) |  |
| Target 139 | *ACPP* | Acid Phosphatase, Prostate | Enzyme that catalyzes the conversion of orthophosphoric monoester to alcohol and orthophosphate. | (Staley et al., 2016) |  |
| Target 140 | *PAPPA* | Pappalysin 1 | Growth factor plays a role in bone formation, inflammation and wound healing. | (Moin et al., 2021) | Filtered out before analysis |
| Target 141 | *PDGFB* | Platelet Derived Growth Factor Subunit B | Activate PDGF receptor tyrosine kinases, which play a role in a wide range of developmental processes. | (Ray et al., 2007) |  |
| Target  142 | *TNFRSF1A* | TNF Receptor  Superfamily Member 1A | Plays a role in cell survival, apoptosis, and inflammation. | (Ray et al., 2007) |  |
| Target 143 | *TNC* | Tenascin C | Implicated in guidance of migrating neurons as well as axons during development, synaptic plasticity, and neuronal regeneration. | (Brosseron et al., 2014) |  |
| Target 144 | *TGFA* | Transforming Growth Factor Alpha | Activates a signaling pathway for cell proliferation, differentiation and development. | (Ray et al., 2007) |  |
| Target 145 | *TGFB1* | Transforming Growth Factor Beta 1 | Regulates cell proliferation, differentiation and growth, and can modulate expression and activation of other growth factors including IFNG and TNF. | (Ray et al., 2007) |  |
| Target 146 | *TIMP1* | TIMP Metallopeptidase Inhibitor 1 | Promote cell proliferation and may also have an anti-apoptotic function. | (Ray et al., 2007) |  |
| Target 147 | *LTA* | Lymphotoxin Alpha | Mediates a large variety of inflammatory, immunostimulatory, and antiviral responses, is involved in the formation of secondary lymphoid organs during development and plays a  role in apoptosis. | (Ray et al., 2007) |  |
| Target 148 | *TNFRSF10D* | TNF Receptor Superfamily Member 10d | Receptor that plays an inhibitory role in TRAIL-induced cell apoptosis. | (Ray et al., 2007) |  |
| Target 149 | *TREM2* | Triggering Receptor Expressed On Myeloid  Cells 2 | Involved in chronic inflammation by triggering the production of constitutive inflammatory cytokines. | (Carmona et al., 2018) |  |
| Target 150 | *VCAM1* | Vascular Cell Adhesion Molecule 1 | Mediates leukocyte-endothelial cell adhesion and signal  transduction, and may play a role in the development of atherosclerosis. | (Zuliani et al., 2008) |  |
| Target 151 | *A2M* | Alpha-2-Macroglobulin | Protease inhibitor and cytokine transporter that inhibit inflammatory cytokines, and disrupts inflammatory cascades. | (Varma et al., 2017) |  |
| Target 152 | *MTHFR* | Methylenetetrahydrofol ate Reductase | Enzyme plays a role in processing amino acids. | (Kamat et al., 2015) |  |
| Target 153 | *TACE* | Tumor Necrosis Factor- alpha-converting enzyme | Enzyme involved in processing of cell adhesion proteins, cytokine and growth factor receptors and epidermal growth factor (EGF) receptor ligands. | (Guedes et al., 2018) |  |
| Target 154 | *TGFB2* | Transforming Growth Factor Beta 2 | Ligands of TGF-beta receptors leading to recruitment of transcription factors that regulate gene expression. | (Guedes et al., 2018) |  |
| Target 155 | *PPARG* | Peroxisome Proliferator  Activated Receptor Gamma | Regulator of adipocyte differentiation. | (Jiang et al., 2008) |  |
| Target 156 | *BACE1* | Beta-Secretase 1 | Transmembrane protease catalyzes the 1st step in the formation of amyloid beta peptide from amyloid precursor protein. | (Wongchitrat et al., 2019) |  |
| Target 157 | *SERPINA1* | Serpin Family A Member 1 | Serine protease inhibitor whose targets include elastase, plasmin, thrombin, trypsin, chymotrypsin, and plasminogen activator. | (Pillai et al., 2019) |  |
| Target 158 | *NLRC3* | NLR Family CARD Domain Containing 3 | Interacts with interferon genes (STING), resulting in disruption of STING-dependent activation of the innate immune response. | (Zha et al., 2020) |  |
| Target 159 | *TLR2* | Toll Like Receptor 2 | Plays a role in pathogen recognition and activation of innate immunity. | (Zhang et al., 2012) |  |
| Target 160 | *S100A9* | S100 Calcium Binding Protein A9 | Regulation of a number of cellular processes such as cell cycle progression and differentiation. | (Cristóvão & Gomes, 2019) |  |
| Target 161 | *F5* | Coagulation Factor V | Cofactor that participates with activated coagulation factor X to activate prothrombin to thrombin. | (Bots et al., 1998) |  |
| Target 162 | *ETS2* | ETS Proto-Oncogene 2, Transcription Factor | Transcription factor which regulates genes involved in development and apoptosis. | (Sacchi et al., 1988) |  |
| Target 163 | *C3AR1* | Complement C3a Receptor 1 | Orphan G protein-coupled receptor for C3a. Activates chemotaxis, granule enzyme release and superoxide anion production. | (Morgan, 2018) |  |
| Target 164 | *ANGPT1* | Angiopoietin 1 | Vascular development and angiogenesis. | (Grammas, 2011) |  |
| Target 165 | *TIMP3* | TIMP Metallopeptidase Inhibitor 3 | Peptidases involved in degradation of the extracellular matrix. | (Grammas, 2011) |  |
| Target  166 | *S100A12* | S100 Calcium Binding  Protein A12 | Plays a prominent role in the regulation of inflammatory  processes and immune response. | (Cristóvão & Gomes, 2019) |  |
| Target 167 | *TET2* | Tet Methylcytosine Dioxygenase 2 | Plays a key role in active DNA demethylation. | (Cochran et al., 2020) |  |
| Target  168 | *GNAS* | GNAS Complex Locus | Activation of adenylyl cyclase & a variety of cellular responses. | (Wan et al., 2020) | Filtered out before analysis |
| Target 169 | *NOTCH3* | Notch Receptor 3 | Key role in neural development. | (Kofler et al., 2015) |  |
| Target 170 | *ABCA7* | ATP Binding Cassette Subfamily A Member 7 | Modulate lipid transport and thereby contribute to phagocytosis. | (Karch et al., 2012) |  |
| Target 171 | *CR1* | Complement C3b/C4b Receptor 1 | Role in immune regulation by generation of regulatory T cells from activated helper T cells. | (Karch et al., 2012) |  |
| Target 172 | *MS4A6A* | Membrane Spanning 4- Domains A6A | Involved in signal transduction as a component of a multimeric receptor complex. | (Karch et al., 2012) |  |
| Target 173 | *MRPL51* | Mitochondrial Ribosomal Protein L51 | Mitochondrial translation and Organelle biogenesis and maintenance. | (Bai et al., 2014) |  |
| Target 174 | *RPL36AL* | Ribosomal Protein L36a Like | Encode ribosomal proteins. | (Bai et al., 2014) |  |
| Target 175 | *RPS25* | Ribosomal Protein L36a Like | Encode ribosomal proteins. | (Bai et al., 2014) |  |
| Target 176 | *CD33* | CD33 Molecule | Plays a role in mediating cell-cell interactions and in maintaining immune cells in a resting state. | (Hu et al., 2014) |  |
| Target 177 | *IL4R* | Interleukin 4 Receptor | Differentiation of Th2 cells. | (Guedes et al., 2018) |  |
| Target 178 | *SOCS3* | Suppressor Of Cytokine Signalling 3 | Negative regulators of cytokine signalling. | (Cao et al., 2018) |  |
| Target 179 | *CXCL2* | C-X-C Motif Chemokine  Ligand 2 | Produced by activated monocytes and neutrophils. Suppresses  hematopoietic progenitor cell proliferation. | (W.-J. Lee et al., 2018) |  |
| Target 180 | *NLRP3* | NLR Family Pyrin Domain Containing 3 | Activator of NF-kappaB signalling, plays a role in the regulation of inflammation, the immune response & apoptosis. | (Saresella et al., 2016) |  |
| Target 181 | *CD2AP* | CD2 Associated Protein | Adapter protein between membrane proteins and the actin cytoskeleton. | (Tao et al., 2017) |  |
| Target 182 | *SORL1* | Sortilin Related Receptor 1 | Regulating the trafficking and recycling of amyloid precursor protein. | (Chou et al., 2016) |  |

**Supplementary References**

Akhtar, W., & Veenstra, G. J. C. (2011). TBP-related factors: A paradigm of diversity in transcription initiation. *Cell & Bioscience*, *1*(1), 23. https://doi.org/10.1186/2045-3701-1-23

Ascolani, A., Balestrieri, E., Minutolo, A., Mosti, S., Spalletta, G., Bramanti, P., Mastino, A., Caltagirone, C., & Macchi, B. (2012). Dysregulated NF-κB Pathway in Peripheral Mononuclear Cells of Alzheimers Disease Patients. *Current Alzheimer Research*, *9*(1), 128–137.

Bai, Z., Stamova, B., Xu, H., Ander, B. P., Wang, J., Jickling, G. C., Zhan, X., Liu, D., Han, G., Jin, L.-W., DeCarli, C., Lei, H., & Sharp, F. R. (2014). Distinctive RNA Expression Profiles in Blood Associated With Alzheimer Disease After Accounting for White Matter Hyperintensities. *Alzheimer Disease & Associated Disorders*, *28*(3), 226–233. https://doi.org/10.1097/WAD.0000000000000022

Baron, B. W., Baron, R. M., & Baron, J. M. (2015). The ITM2B (BRI2) gene is a target of BCL6 repression: Implications for lymphomas and neurodegenerative diseases. *Biochimica et Biophysica Acta (BBA) - Molecular Basis of Disease*, *1852*(5), 742–748. https://doi.org/10.1016/j.bbadis.2014.12.018

Bednarska-Makaruk, M., Graban, A., Wiśniewska, A., Łojkowska, W., Bochyńska, A., Gugała-Iwaniuk, M., Sławińska, K., Ługowska, A., Ryglewicz, D., & Wehr, H. (2017). Association of adiponectin, leptin and resistin with inflammatory markers and obesity in dementia. *Biogerontology*, *18*(4), 561–580. https://doi.org/10.1007/s10522-017-9701-0

Bots, M. L., Breteler, M. M. B., Kooten, F. van, Haverkate, F., Meijer, P., Koudstaal, P. J., Grobbee, D. E., & Kluft, C. (1998). Coagulation and Fibrinolysis Markers and Risk of Dementia. *Pathophysiology of Haemostasis and Thrombosis*, *28*(3–4), 216–222. https://doi.org/10.1159/000022433

Bouzid, H., Belk, J., Jan, M., Qi, Y., Sarnowski, C., Wirth, S., Ma, L., Chrostek, M., Ahmad, H., Nachun, D., Yao, W., Beiser, A., Bick, A. G., Bis, J., Fornage, M., Longstreth, W. T., Lopez, O., Nataranjan, P., Psaty, B., … Jaiswal, S. (2021). Clonal Hematopoiesis is Associated with Reduced Risk of Alzheimer’s Disease. *Blood*, *138*, 5. https://doi.org/10.1182/blood-2021-151064

Brosseron, F., Krauthausen, M., Kummer, M., & Heneka, M. T. (2014). Body Fluid Cytokine Levels in Mild Cognitive Impairment and Alzheimer’s Disease: A Comparative Overview. *Molecular Neurobiology*, *50*(2), 534–544. https://doi.org/10.1007/s12035-014-8657-1

Buerger, K., Uspenskaya, O., Hartmann, O., Hansson, O., Minthon, L., Blennow, K., Moeller, H.-J., Teipel, S. J., Ernst, A., Bergmann, A., & Hampel, H. (2010). Prediction of Alzheimer’s Disease Using Midregional Proadrenomedullin and Midregional Proatrial Natriuretic Peptide: A Retrospective Analysis of 134 Patients With Mild Cognitive Impairment. *The Journal of Clinical Psychiatry*, *71*(4), 15017. https://doi.org/10.4088/JCP.09m05872oli

Cao, L., Wang, Z., & Wan, W. (2018). Suppressor of Cytokine Signaling 3: Emerging Role Linking Central Insulin Resistance and Alzheimer’s Disease. *Frontiers in Neuroscience*, *12*. https://www.frontiersin.org/article/10.3389/fnins.2018.00417

Carmona, S., Zahs, K., Wu, E., Dakin, K., Bras, J., & Guerreiro, R. (2018). The role of TREM2 in Alzheimer’s disease and other neurodegenerative disorders. *The Lancet Neurology*, *17*(8), 721–730. https://doi.org/10.1016/S1474-4422(18)30232-1

Chiba, T., Yamada, M., Sasabe, J., Terashita, K., Shimoda, M., Matsuoka, M., & Aiso, S. (2009). Amyloid-β causes memory impairment by disturbing the JAK2/STAT3 axis in hippocampal neurons. *Molecular Psychiatry*, *14*(2), 206–222. https://doi.org/10.1038/mp.2008.105

Chou, C.-T., Liao, Y.-C., Lee, W.-J., Wang, S.-J., & Fuh, J.-L. (2016). SORL1 gene, plasma biomarkers, and the risk of Alzheimer’s disease for the Han Chinese population in Taiwan. *Alzheimer’s Research & Therapy*, *8*(1), 53. https://doi.org/10.1186/s13195-016-0222-x

Cochran, J. N., Geier, E. G., Bonham, L. W., Newberry, J. S., Amaral, M. D., Thompson, M. L., Lasseigne, B. N., Karydas, A. M., Roberson, E. D., Cooper, G. M., Rabinovici, G. D., Miller, B. L., Myers, R. M., & Yokoyama, J. S. (2020). Non-coding and Loss-of-Function Coding Variants in TET2 are Associated with Multiple Neurodegenerative Diseases. *The American Journal of Human Genetics*, *106*(5), 632–645. https://doi.org/10.1016/j.ajhg.2020.03.010

Craig-Schapiro, R., Perrin, R. J., Roe, C. M., Xiong, C., Carter, D., Cairns, N. J., Mintun, M. A., Peskind, E. R., Li, G., Galasko, D. R., Clark, C. M., Quinn, J. F., D’Angelo, G., Malone, J. P., Townsend, R. R., Morris, J. C., Fagan, A. M., & Holtzman, D. M. (2010). YKL-40: A Novel Prognostic Fluid Biomarker for Preclinical Alzheimer’s Disease. *Biological Psychiatry*, *68*(10), 903–912. https://doi.org/10.1016/j.biopsych.2010.08.025

Cristóvão, J. S., & Gomes, C. M. (2019). S100 Proteins in Alzheimer’s Disease. *Frontiers in Neuroscience*, *13*. https://www.frontiersin.org/article/10.3389/fnins.2019.00463

Das, R., & Chinnathambi, S. (2019). Microglial priming of antigen presentation and adaptive stimulation in Alzheimer’s disease. *Cellular and Molecular Life Sciences*, *76*(19), 3681–3694. https://doi.org/10.1007/s00018-019-03132-2

Du, Y., Xu, J.-T., Jin, H.-N., Zhao, R., Zhao, D., Du, S.-H., Xue, Y., Xie, X.-L., & Wang, Q. (2017). Increased cerebral expressions of MMPs, CLDN5, OCLN, ZO1 and AQPs are associated with brain edema following fatal heat stroke. *Scientific Reports*, *7*, 1691. https://doi.org/10.1038/s41598-017-01923-w

Gatta, L., Cardinale, A., Wannenes, F., Consoli, C., Armani, A., Molinari, F., Mammi, C., Stocchi, F., Torti, M., Rosano, G. M. C., & Fini, M. (2009). Peripheral blood mononuclear cells from mild cognitive impairment patients show deregulation of Bax and Sod1 mRNAs. *Neuroscience Letters*, *453*(1), 36–40. https://doi.org/10.1016/j.neulet.2009.02.003

Grammas, P. (2011). Neurovascular dysfunction, inflammation and endothelial activation: Implications for the pathogenesis of Alzheimer’s disease. *Journal of Neuroinflammation*, *8*(1), 26. https://doi.org/10.1186/1742-2094-8-26

Guedes, J. R., Lao, T., Cardoso, A. L., & El Khoury, J. (2018). Roles of Microglial and Monocyte Chemokines and Their Receptors in Regulating Alzheimer’s Disease-Associated Amyloid-β and Tau Pathologies. *Frontiers in Neurology*, *9*. https://www.frontiersin.org/article/10.3389/fneur.2018.00549

Haddick, P. C. G., Larson, J. L., Rathore, N., Bhangale, T. R., Phung, Q. T., Srinivasan, K., Hansen, D. V., Lill, J. R., Pericak-Vance, M. A., Haines, J., Farrer, L. A., Kauwe, J. S., Schellenberg, G. D., Cruchaga, C., Goate, A. M., Behrens, T. W., Watts, R. J., Graham, R. R., Kaminker, J. S., & van der Brug, M. (2017). A common variant of IL-6R is associated with elevated IL-6 pathway activity in Alzheimer’s disease brains. *Journal of Alzheimer’s Disease : JAD*, *56*(3), 1037–1054. https://doi.org/10.3233/JAD-160524

Hu, N., Tan, M.-S., Sun, L., Jiang, T., Wang, Y.-L., Tan, L., Zhang, W., Yu, J.-T., & Tan, L. (2014). Decreased expression of CD33 in peripheral mononuclear cells of Alzheimer’s disease patients. *Neuroscience Letters*, *563*, 51–54. https://doi.org/10.1016/j.neulet.2014.01.004

Jiang, Q., Heneka, M., & Landreth, G. E. (2008). The Role of Peroxisome Proliferator-Activated Receptor-γ PPARγ) in Alzheimer’s Disease. *CNS Drugs*, *22*(1), 1–14. https://doi.org/10.2165/00023210-200822010-00001

Julian, A., Rioux-Bilan, A., Ragot, S., Krolak-Salmon, P., Berrut, G., Dantoine, T., Hommet, C., Hanon, O., Page, G., & Paccalin, M. (2018). Blood Inflammatory Mediators and Cognitive Decline in Alzheimer’s Disease: A Two Years Longitudinal Study. *Journal of Alzheimer’s Disease*, *63*(1), 87–92. https://doi.org/10.3233/JAD-171131

Kalinowska, A., & Losy, J. (2006). PECAM-1, a key player in neuroinflammation. *European Journal of Neurology*, *13*(12), 1284–1290. https://doi.org/10.1111/j.1468-1331.2006.01640.x

Kamat, P. K., Vacek, J. C., Kalani, A., & Tyagi, N. (2015). Homocysteine Induced Cerebrovascular Dysfunction: A Link to Alzheimer’s Disease Etiology. *The Open Neurology Journal*, *9*(1). https://doi.org/10.2174/1874205X01509010009

Kamboh, M. I., Minster, R. L., Kenney, M., Ozturk, A., Desai, P. P., Kammerer, C. M., & DeKosky, S. T. (2006). Alpha-1-antichymotrypsin (ACT or SERPINA3) polymorphism may affect age-at-onset and disease duration of Alzheimer’s disease. *Neurobiology of Aging*, *27*(10), 1435–1439. https://doi.org/10.1016/j.neurobiolaging.2005.07.015

Karch, C. M., Jeng, A. T., Nowotny, P., Cady, J., Cruchaga, C., & Goate, A. M. (2012). Expression of Novel Alzheimer’s Disease Risk Genes in Control and Alzheimer’s Disease Brains. *PLOS ONE*, *7*(11), e50976. https://doi.org/10.1371/journal.pone.0050976

Kofler, N. M., Cuervo, H., Uh, M. K., Murtomäki, A., & Kitajewski, J. (2015). Combined deficiency of Notch1 and Notch3 causes pericyte dysfunction, models CADASIL, and results in arteriovenous malformations. *Scientific Reports*, *5*, 16449. https://doi.org/10.1038/srep16449

Lee, T.-S., Goh, L., Chong, M. S., Chua, S. M., Chen, G. B., Feng, L., Lim, W. S., Chan, M., Ng, T. P., & Ranga Krishnan, K. (2012). Downregulation of TOMM40 expression in the blood of Alzheimer disease subjects compared with matched controls. *Journal of Psychiatric Research*, *46*(6), 828–830. https://doi.org/10.1016/j.jpsychires.2012.03.006

Lee, W.-J., Liao, Y.-C., Wang, Y.-F., Lin, I.-F., Wang, S.-J., & Fuh, J.-L. (2018). Plasma MCP-1 and Cognitive Decline in Patients with Alzheimer’s Disease and Mild Cognitive Impairment: A Two-year Follow-up Study. *Scientific Reports*, *8*, 1280. https://doi.org/10.1038/s41598-018-19807-y

Lin, E., Kuo, P.-H., Liu, Y.-L., Yang, A. C., & Tsai, S.-J. (2019). Association and Interaction Effects of Interleukin-12 Related Genes and Physical Activity on Cognitive Aging in Old Adults in the Taiwanese Population. *Frontiers in Neurology*, *10*, 1065. https://doi.org/10.3389/fneur.2019.01065

Ling, C., Fangyu, D., Wanhua, H., Kelong, C., zhimin, W., yuting, Z., & rong, Z. (2016). DNMT3A rs1550117 Polymorphism is Associated With Late-Onset Alzheimer’s Disease in a Chinese Population. *American Journal of Alzheimer’s Disease & Other Dementias®*, *31*(3), 278–281. https://doi.org/10.1177/1533317515603688

Liu, Y., Yu, J.-T., Zhang, W., Zong, Y., Lu, R.-C., Zhou, J., & Tan, L. (2014). Interleukin-23 receptor polymorphisms are associated with Alzheimer’s disease in Han Chinese. *Journal of Neuroimmunology*, *271*(1), 43–48. https://doi.org/10.1016/j.jneuroim.2014.03.013

Mahinrad, S., Bulk, M., van der Velpen, I., Mahfouz, A., van Roon-Mom, W., Fedarko, N., Yasar, S., Sabayan, B., van Heemst, D., & van der Weerd, L. (2018). Natriuretic Peptides in Post-mortem Brain Tissue and Cerebrospinal Fluid of Non-demented Humans and Alzheimer’s Disease Patients. *Frontiers in Neuroscience*, *12*, 864. https://doi.org/10.3389/fnins.2018.00864

Manavalan, A., Mishra, M., Feng, L., Sze, S. K., Akatsu, H., & Heese, K. (2013). Brain site-specific proteome changes in aging-related dementia. *Experimental & Molecular Medicine*, *45*(9), e39–e39. https://doi.org/10.1038/emm.2013.76

Moin, A. S. M., Kahal, H., Al-Qaissi, A., Kumar, N., Sathyapalan, T., Atkin, S. L., & Butler, A. E. (2021). Amyloid-related protein changes associated with dementia differ according to severity of hypoglycemia. *BMJ Open Diabetes Research & Care*, *9*(1), e002211. https://doi.org/10.1136/bmjdrc-2021-002211

Morgan, B. P. (2018). Complement in the pathogenesis of Alzheimer’s disease. *Seminars in Immunopathology*, *40*(1), 113–124. https://doi.org/10.1007/s00281-017-0662-9

Nho, K., Kim, S., Horgusluoglu, E., Risacher, S. L., Shen, L., Kim, D., Lee, S., Foroud, T., Shaw, L. M., Trojanowski, J. Q., Aisen, P. S., Petersen, R. C., Jack, C. R., Weiner, M. W., Green, R. C., Toga, A. W., Saykin, A. J., & for the Alzheimer’s Disease Neuroimaging Initiative (ADNI). (2017). Association analysis of rare variants near the APOE region with CSF and neuroimaging biomarkers of Alzheimer’s disease. *BMC Medical Genomics*, *10*(1), 29. https://doi.org/10.1186/s12920-017-0267-0

O’Bryant, S. E., Xiao, G., Barber, R., Huebinger, R., Wilhelmsen, K., Edwards, M., Graff-Radford, N., Doody, R., Diaz-Arrastia, R., Consortium, for the T. A. R. & C., & Initiative, for the A. D. N. (2011). A Blood-Based Screening Tool for Alzheimer’s Disease That Spans Serum and Plasma: Findings from TARC and ADNI. *PLOS ONE*, *6*(12), e28092. https://doi.org/10.1371/journal.pone.0028092

Palmer, J. C., Barker, R., Kehoe, P. G., & Love, S. (2012). Endothelin-1 is elevated in Alzheimer’s disease and upregulated by amyloid-β. *Journal of Alzheimer’s Disease: JAD*, *29*(4), 853–861. https://doi.org/10.3233/JAD-2012-111760

Park, C.-W., & Ryu, K.-Y. (2014). Cellular ubiquitin pool dynamics and homeostasis. *BMB Reports*, *47*(9), 475–482. https://doi.org/10.5483/BMBRep.2014.47.9.128

Penna, I., Vella, S., Gigoni, A., Russo, C., Cancedda, R., & Pagano, A. (2011). Selection of Candidate Housekeeping Genes for Normalization in Human Postmortem Brain Samples. *International Journal of Molecular Sciences*, *12*(9), 5461–5470. https://doi.org/10.3390/ijms12095461

Pey, P., Pearce, R. K., Kalaitzakis, M. E., Griffin, W. S. T., & Gentleman, S. M. (2014). Phenotypic profile of alternative activation marker CD163 is different in Alzheimer’s and Parkinson’s disease. *Acta Neuropathologica Communications*, *2*, 21. https://doi.org/10.1186/2051-5960-2-21

Pillai, J. A., Maxwell, S., Bena, J., Bekris, L. M., Rao, S. M., Chance, M., Lamb, B. T., & Leverenz, J. B. (2019). Key inflammatory pathway activations in the MCI stage of Alzheimer’s disease. *Annals of Clinical and Translational Neurology*, *6*(7), 1248–1262. https://doi.org/10.1002/acn3.50827

Qian, W., & Liu, F. (2014). Regulation of alternative splicing of tau exon 10. *Neuroscience Bulletin*, *30*(2), 367–377. https://doi.org/10.1007/s12264-013-1411-2

Rangaraju, S., Dammer, E. B., Raza, S. A., Gao, T., Xiao, H., Betarbet, R., Duong, D. M., Webster, J. A., Hales, C. M., Lah, J. J., Levey, A. I., & Seyfried, N. T. (2018). Quantitative proteomics of acutely-isolated mouse microglia identifies novel immune Alzheimer’s disease-related proteins. *Molecular Neurodegeneration*, *13*(1), 34. https://doi.org/10.1186/s13024-018-0266-4

Ray, S., Britschgi, M., Herbert, C., Takeda-Uchimura, Y., Boxer, A., Blennow, K., Friedman, L. F., Galasko, D. R., Jutel, M., Karydas, A., Kaye, J. A., Leszek, J., Miller, B. L., Minthon, L., Quinn, J. F., Rabinovici, G. D., Robinson, W. H., Sabbagh, M. N., So, Y. T., … Wyss-Coray, T. (2007). Classification and prediction of clinical Alzheimer’s diagnosis based on plasma signaling proteins. *Nature Medicine*, *13*(11), 1359–1362. https://doi.org/10.1038/nm1653

Rybak-Wolf, A., & Plass, M. (2021). RNA Dynamics in Alzheimer’s Disease. *Molecules*, *26*(17), 5113. https://doi.org/10.3390/molecules26175113

Sacchi, N., Nalbantoglu, J., Sergovich, F. R., & Papas, T. S. (1988). Human ETS2 gene on chromosome 21 is not rearranged in Alzheimer disease. *Proceedings of the National Academy of Sciences*, *85*(20), 7675–7679. https://doi.org/10.1073/pnas.85.20.7675

Saresella, M., La Rosa, F., Piancone, F., Zoppis, M., Marventano, I., Calabrese, E., Rainone, V., Nemni, R., Mancuso, R., & Clerici, M. (2016). The NLRP3 and NLRP1 inflammasomes are activated in Alzheimer’s disease. *Molecular Neurodegeneration*, *11*(1), 23. https://doi.org/10.1186/s13024-016-0088-1

Slusher, A. L., Zúñiga, T. M., & Acevedo, E. O. (2019). Inflamm-Aging Is Associated with Lower Plasma PTX3 Concentrations and an Impaired Capacity of PBMCs to Express hTERT following LPS Stimulation. *Mediators of Inflammation*, *2019*, e2324193. https://doi.org/10.1155/2019/2324193

Spindler, M., Niebler, R., Remkes, H., Horn, M., Lanz, T., & Neubauer, S. (2002). Mitochondrial creatine kinase is critically necessary for normal myocardial high-energy phosphate metabolism. *American Journal of Physiology-Heart and Circulatory Physiology*, *283*(2), H680–H687. https://doi.org/10.1152/ajpheart.00800.2001

Staley, L. A., Ebbert, M. T. W., Bunker, D., Bailey, M., Ridge, P. G., Goate, A. M., & Kauwe, J. S. K. (2016). Variants in ACPP are associated with cerebrospinal fluid Prostatic Acid Phosphatase levels. *BMC Genomics*, *17*(Suppl 3), 439. https://doi.org/10.1186/s12864-016-2787-y

Swaminathan, G., Zhu, W., & Plowey, E. D. (2016). BECN1/Beclin 1 sorts cell-surface APP/amyloid β precursor protein for lysosomal degradation. *Autophagy*, *12*(12), 2404–2419. https://doi.org/10.1080/15548627.2016.1234561

Tao, Q.-Q., Liu, Z.-J., Sun, Y.-M., Li, H.-L., Yang, P., Liu, D.-S., Jiang, B., Li, X.-Y., Xu, J.-F., & Wu, Z.-Y. (2017). Decreased gene expression of CD2AP in Chinese patients with sporadic Alzheimer’s disease. *Neurobiology of Aging*, *56*, 212.e5-212.e10. https://doi.org/10.1016/j.neurobiolaging.2017.03.013

Turner, R. J., & Sharp, F. R. (2016). Implications of MMP9 for Blood Brain Barrier Disruption and Hemorrhagic Transformation Following Ischemic Stroke. *Frontiers in Cellular Neuroscience*, *10*. https://www.frontiersin.org/article/10.3389/fncel.2016.00056

Van Eldik, L. J., Carrillo, M. C., Cole, P. E., Feuerbach, D., Greenberg, B. D., Hendrix, J. A., Kennedy, M., Kozauer, N., Margolin, R. A., Molinuevo, J. L., Mueller, R., Ransohoff, R. M., Wilcock, D. M., Bain, L., & Bales, K. (2016). The roles of inflammation and immune mechanisms in Alzheimer’s disease. *Alzheimer’s & Dementia : Translational Research & Clinical Interventions*, *2*(2), 99–109. https://doi.org/10.1016/j.trci.2016.05.001

Varma, V., Varma, S., An, Y., Hohman, T., Seddighi, S., Casanova, R., Beri, A., Dammer, E., Seyfried, N., Pletnikova, O., Moghekar, A., Wilson, M., Lah, J., O’Brien, R., Levey, A., Troncoso, J., Albert, M., & Thambisetty, M. (2017). Alpha-2 macroglobulin in Alzheimer’s disease: A marker of neuronal injury through the RCAN1 pathway. *Molecular Psychiatry*, *22*(1), 13–23. https://doi.org/10.1038/mp.2016.206

Wan, Y.-W., Al-Ouran, R., Mangleburg, C. G., Perumal, T. M., Lee, T. V., Allison, K., Swarup, V., Funk, C. C., Gaiteri, C., Allen, M., Wang, M., Neuner, S. M., Kaczorowski, C. C., Philip, V. M., Howell, G. R., Martini-Stoica, H., Zheng, H., Mei, H., Zhong, X., … Logsdon, B. A. (2020). Meta-Analysis of the Alzheimer’s Disease Human Brain Transcriptome and Functional Dissection in Mouse Models. *Cell Reports*, *32*(2), 107908. https://doi.org/10.1016/j.celrep.2020.107908

Wong, J. (2013). Altered Expression of RNA Splicing Proteins in Alzheimer’s Disease Patients: Evidence from Two Microarray Studies. *Dementia and Geriatric Cognitive Disorders EXTRA*, *3*(1), 74–85. https://doi.org/10.1159/000348406

Wongchitrat, P., Pakpian, N., Kitidee, K., Phopin, K., Dharmasaroja, P. A., & Govitrapong, P. (2019). Alterations in the Expression of Amyloid Precursor Protein Cleaving Enzymes mRNA in Alzheimer Peripheral Blood. *Current Alzheimer Research*, *16*(1), 29–38. https://doi.org/10.2174/1567205015666181109103742

Wright, K. J., Baye, L. M., Olivier-Mason, A., Mukhopadhyay, S., Sang, L., Kwong, M., Wang, W., Pretorius, P. R., Sheffield, V. C., Sengupta, P., Slusarski, D. C., & Jackson, P. K. (2011). An ARL3–UNC119–RP2 GTPase cycle targets myristoylated NPHP3 to the primary cilium. *Genes & Development*, *25*(22), 2347–2360. https://doi.org/10.1101/gad.173443.111

Yang, J., Kou, J., Lalonde, R., & Fukuchi, K. (2017). Intracranial IL-17A overexpression decreases cerebral amyloid angiopathy by upregulation of ABCA1 in an animal model of Alzheimer’s disease. *Brain, Behavior, and Immunity*, *65*, 262–273. https://doi.org/10.1016/j.bbi.2017.05.012

Yun, H.-M., Kim, J. A., Hwang, C. J., Jin, P., Baek, M. K., Lee, J. M., Hong, J. E., Lee, S. M., Han, S. B., Oh, K. W., Choi, D. Y., Yoon, D. Y., & Hong, J. T. (2015). Neuroinflammatory and Amyloidogenic Activities of IL-32β in Alzheimer’s Disease. *Molecular Neurobiology*, *52*(1), 341–352. https://doi.org/10.1007/s12035-014-8860-0

Zha, L., Yu, Z., Fang, J., Zhou, L., Guo, W., & Zhou, J. (2020). NLRC3 Delays the Progression of AD in APP/PS1 Mice via Inhibiting PI3K Activation. *Oxidative Medicine and Cellular Longevity*, *2020*, 5328031. https://doi.org/10.1155/2020/5328031

Zhang, W., Wang, L.-Z., Yu, J.-T., Chi, Z.-F., & Tan, L. (2012). Increased expressions of TLR2 and TLR4 on peripheral blood mononuclear cells from patients with Alzheimer’s disease. *Journal of the Neurological Sciences*, *315*(1), 67–71. https://doi.org/10.1016/j.jns.2011.11.032

Zheng, C., Zhou, X.-W., & Wang, J.-Z. (2016). The dual roles of cytokines in Alzheimer’s disease: Update on interleukins, TNF-α, TGF-β and IFN-γ. *Translational Neurodegeneration*, *5*, 7. https://doi.org/10.1186/s40035-016-0054-4

Zuliani, G., Cavalieri, M., Galvani, M., Passaro, A., Munari, M. R., Bosi, C., Zurlo, A., & Fellin, R. (2008). Markers of endothelial dysfunction in older subjects with late onset Alzheimer’s disease or vascular dementia. *Journal of the Neurological Sciences*, *272*(1), 164–170. https://doi.org/10.1016/j.jns.2008.05.020
