# Supplementary material for: Alzheimer's Disease Blood Biomarkers Associated With Neuroinflammation as Therapeutic Targets for Early Personalized Intervention
Source: Front Digit Health. 2022 Jul 11;4:875895. doi: 10.3389/fdgth.2022.875895 (PMC9309434; doi:10.3389/fdgth.2022.875895)
Supplement: Supplementary file 2 [file Table_2.docx]

**Supplementary Table 2: Highest prediction accuracy for National Neuroscience Institute (NNI) data using differentially expressed genes as predictive features**

| Comparison | Highest Prediction Accuracy  (mean % ± s.e.) | *p*-value |
| --- | --- | --- |
| CN vs. MCI | 60.25 ± 0.25 | 7.61e-12 *** |
| CN vs. AD | 56.25 ± 0.67 | 3.26e-06 *** |
| MCI vs. AD | 73.95 ± 0.26 | 5.92e-15 *** |

Supplementary Table 2 shows the mean and standard error (s.e.) of the highest prediction percent accuracy when random forest models constructed using differentially expressed genes shown in Table 2 are used to classify subjects between pairwise comparisons of cognitively normal (CN) control vs. mild cognitive impairment (MCI), CN vs. Alzheimer’s disease (AD), and MCI vs. AD subjects from NNI. *** indicates *p* < 0.001 according to a one-tailed t-test with the alternative hypothesis that the mean highest prediction percent accuracy is greater than 50%.
