# Supplementary material for: Alzheimer's Disease Blood Biomarkers Associated With Neuroinflammation as Therapeutic Targets for Early Personalized Intervention
Source: Front Digit Health. 2022 Jul 11;4:875895. doi: 10.3389/fdgth.2022.875895 (PMC9309434; doi:10.3389/fdgth.2022.875895)
Supplement: Supplementary file 3 [file Table_3.docx]

**Supplementary Table 3: Highest prediction accuracies (mean ± standard error) for differentially expressed genes from Li et al. (2017), Li et al. (2018), and from our study as predictive features as evaluated on ADNI data**

| Comparison | Li et al. (2017) | Li et al. (2018) | This Study |
| --- | --- | --- | --- |
| CN vs. MCI | 53.01 ± 0.21 | 53.07 ± 0.27 | 59.55 ± 0.24 |
| CN vs. AD | 55.58 ± 0.24 | 57.60 ± 0.10 | 55.96 ± 0.13 |
| MCI vs. AD | 47.50 ± 0.13 | 54.94 ± 0.12 | 56.65 ± 0.09 |

Supplementary Table 3 shows the mean and standard error (s.e.) of the highest prediction percent accuracy when random forest models constructed using differentially expressed genes from either Li et al. (2017), Li et al. (2018), or DEGs from Boruta shown in Table 2 are used to classify subjects between pairwise comparisons of cognitively normal (CN) control vs. mild cognitive impairment (MCI), CN vs. Alzheimer’s disease (AD), and MCI vs. AD from ADNI. For genes from literature, classification models were constructed using genes listed in each study, for which expression values were also available in the ADNI dataset. Genes included from Li et al. (2017): *ATP5I*, *GTF2B*, *DNAJA1*, *NDUFA1*, *RPA3*, *RPL26*, *RPS27A*, *MRPL33*, *MRPL51*, *CWC15*, *ZC3H15*, *POMP*, *RPF1*, *IGBP1*, *ZMAT2*, *DPM1*, *ANAPC13*, *DBI*, *SNTB2* and *NOP10*. Genes included from Li et al. (2018): *MRPL51*, *RPL36AL*, *CETN2*, *AHSA1* and *ING3*.
