# Supplementary material for: Alzheimer's Disease Blood Biomarkers Associated With Neuroinflammation as Therapeutic Targets for Early Personalized Intervention
Source: Front Digit Health. 2022 Jul 11;4:875895. doi: 10.3389/fdgth.2022.875895 (PMC9309434; doi:10.3389/fdgth.2022.875895)
Supplement: Supplementary file 4 [file Data_Sheet_1.docx]

**
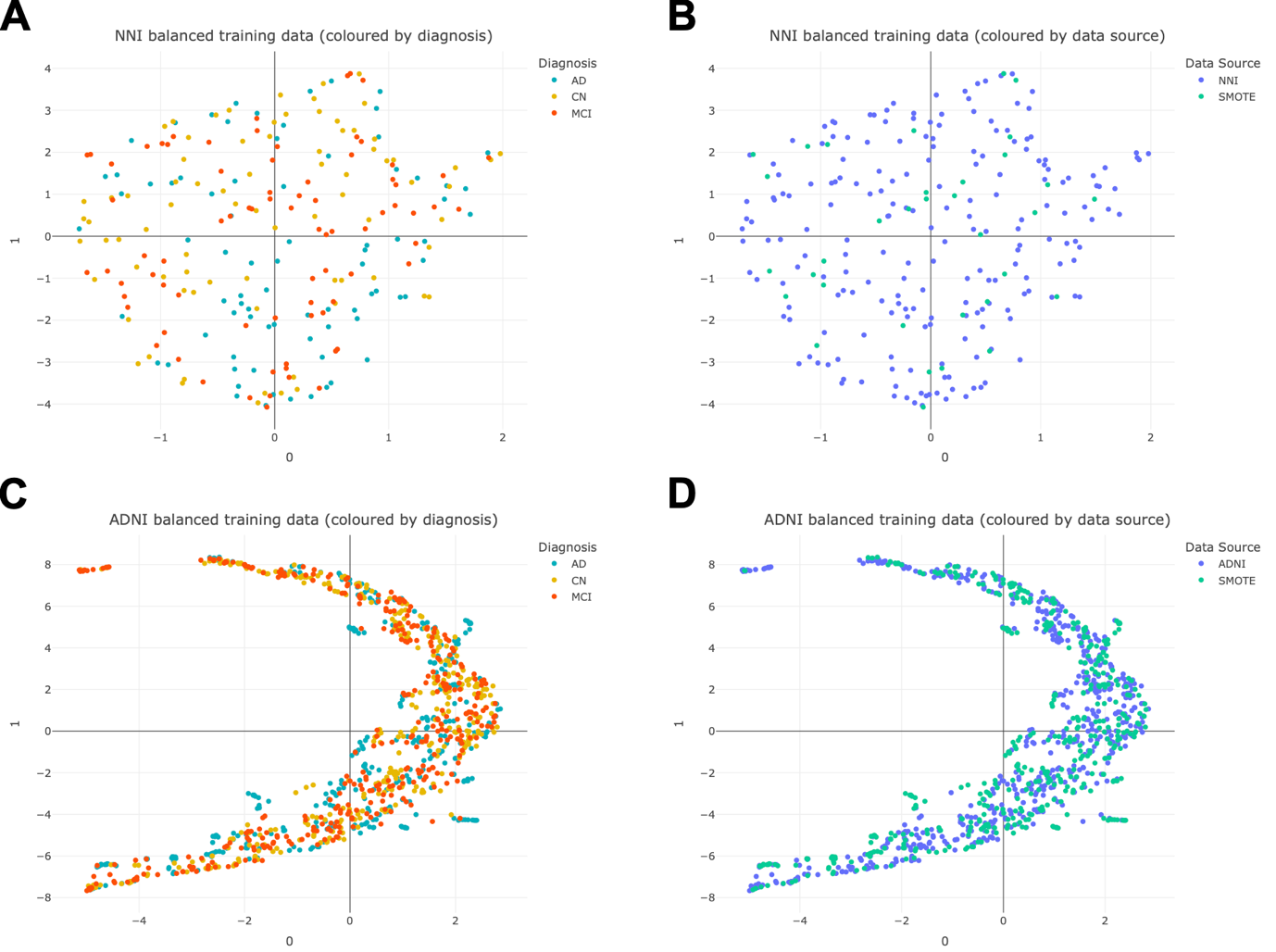
**

**Supplementary Figure 1: Uniform manifold approximation and projection plots for training data from the National Neuroscience Institute (NNI) and the Alzheimer’s Disease Neuroimaging Initiative (ADNI).** (A-B) NNI training data, coloured by diagnosis in (A) and by data source (originally from NNI or from applying Synthetic Minority Oversampling Technique (SMOTE)) in (B). (C-D) ADNI training data, coloured by diagnosis in (C), and by data source (originally from ADNI or from SMOTE) in (D). CN - cognitively normal; MCI - mild cognitive impairment; AD - Alzheimer’s Disease.
