# Supplementary figures and images for: Alzheimer's Disease Blood Biomarkers Associated With Neuroinflammation as Therapeutic Targets for Early Personalized Intervention
Source: Front Digit Health. 2022 Jul 11;4:875895. doi: 10.3389/fdgth.2022.875895 (PMC9309434; doi:10.3389/fdgth.2022.875895)

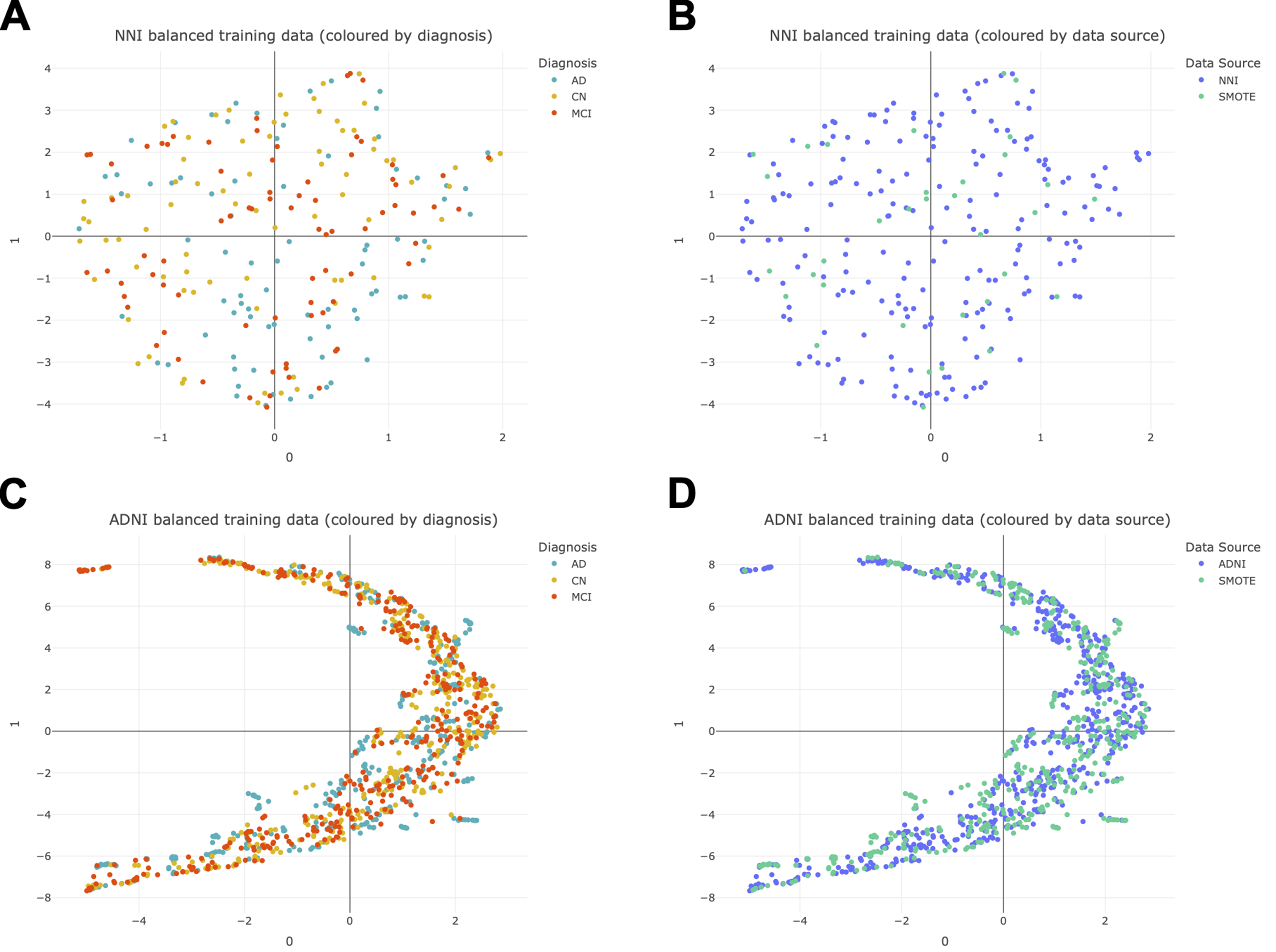

Supplement: Supplementary file 5 [file Image_1.tiff]
